# A living mapping review for COVID-19 funded research projects: six-month update

**DOI:** 10.12688/wellcomeopenres.16259.3

**Published:** 2021-04-01

**Authors:** Alice Norton, Adrian Bucher, Emilia Antonio, Nicole Advani, Henrike Grund, Sheila Mburu, Emma Clegg, Marguerite Gollish, Nusrat Jabin, Laura Scott, Genevieve Boily-Larouche, A. Morgan Lay, Gail Carson, Marta Tufet Bayona

**Affiliations:** 1 UK Collaborative on Development Research, London, UK; 2 GloPID-R Secretariat, Centre for Tropical Medicine and Global Health, University of Oxford, Oxford, UK; 3 Centre for Tropical Medicine and Global Health, University of Oxford, Oxford, UK; 4 Institute of Infection and Immunity, Canadian Institutes of Health Research, Hamilton, Canada; 5 Institute of Population and Public Health, Canadian Institutes of Health Research, Toronto, Canada

**Keywords:** Living systematic review, COVID-19, Coronavirus, research funding, coordination, global health policy

## Abstract

**Background: **The coronavirus disease 2019 (COVID-19) has resulted in an unprecedented research response, demonstrating exceptional examples of rapid research and collaboration. There is however a need for greater coordination, with limited resources and the shifting global nature of the pandemic resulting in a proliferation of research projects underpowered and unable to achieve their aims.

**Methods: **The UK Collaborative on Development Research (UKCDR) and Global Research Collaboration for Infectious Disease Preparedness (GloPID-R), two funder coordination groups have collaborated to develop a live database of funded research projects across the world relating to COVID-19. Drawing data continually from their members and further global funding bodies, as of 15 ^th^ January 2021 the database contains 7,778 projects, funded by 101 funders, taking place across 136 countries representing an investment of at least $3.8 billion. To our knowledge it is one of the most comprehensive databases. The database is aligned to the World Health Organisation (WHO) Global Research Roadmap: 2019 Novel Coronavirus. It is being used by the WHO, governments and multi-lateral policy makers, research funders and researchers.

This living mapping review aims to supplement the database by providing an open accessible and frequently updated resource summarising the characteristics of the COVID-19 funded research portfolio. Both descriptive and thematic analysis will be presented and updated frequently to aid interpretation of the global COVID-19 funded research portfolio.

**Results: **In this version three analysis we provide an updated detailed descriptive analysis of the database (three months after version two) and focus our thematic analysis on research gaps, research areas in need of coordination, study populations and research locations (with a focus on resource-limited countries).

**Conclusions:** As the global funding response to COVID-19 plateaus, this living mapping review helps both funders and researchers to prioritise resources to areas where there is continued unmet research need.

## Introduction

Researchers and research funders in global health have been preparing for a pandemic such as that caused by severe acute respiratory syndrome coronavirus 2 (SARS-CoV-2) for decades; however, the urgency and global scale of the research needs and response have been difficult to respond to and coordinate. Research funders have rapidly supported repurposing of existing studies and launched rapid funding calls to support research into the most pressing needs. Lessons in expediting research have been learnt from undertaking research in the recent Democratic Republic of Congo Ebola outbreaks and West Africa Ebola, Zika and SARS epidemics, however the truly global nature of the coronavirus disease 2019 (COVID-19) pandemic has led to unprecedented needs and challenges for coordination.

The World Health Organisation (WHO) triggered a rapid response, building on the R&D Blueprint ^1^, and co-organised the Global Research and Innovation Forum: Towards a Research Roadmap for the 2019 Novel Coronavirus meeting with the Global Research Collaboration for Infectious Disease Preparedness (GloPID-R) on February 11–12, 2020 at which over 400 global experts identified research priorities for COVID-19. In March 2020, the WHO released the WHO Coordinated Global Research Roadmap: 2019 Novel Coronavirus (WHO Roadmap) ^2^ to coordinate and accelerate the global research response against the identified priorities. The WHO Roadmap is an unprecedented galvanizing document for global research collaboration. This project builds on this to help shepherd the global response.

In a joint effort to further coordinate and synergise the funding of research to address the WHO Roadmap identified priority areas, the UK Collaborative on Development Research (UKCDR) partnered with GloPID-R to launch the COVID-19 Research Project Tracker ^3^ (the tracker) on April 3, 2020. The tracker is a live database of funded research projects across the world related to the current COVID-19 pandemic. It includes both newly funded research projects and repurposed research projects across all disciplines and categorises them against the mid- to long-term research and development priorities and sub-priorities identified in the WHO Roadmap. Mapping of projects as soon as funding is announced allows visibility of the funded research portfolio well in advance of any outputs such as publications. To date, the database has been accessed over 25 thousand times and we have had active engagement (beyond data entry) with over 20 funders, the WHO (and associated COVID-19 research priority area groups) and research groups regarding its use and the living mapping review. The data is also being extracted by several other funding tracking tools (including Europe Pub Med Central).

The UKCDR Epidemics Preparedness and Response Funders Group ^4^ and GloPID-R Funders groups have each been meeting frequently during the pandemic to strengthen UK and global COVID-19 research funding coordination activities respectively. Their work is informed by the data and analysis from the tracker. Several members of both organisations have recently launched calls for research on COVID-19 in low and middle-income country (LMIC) settings. There is a particular concern that due to the resource limitations in LMICs an uncoordinated approach could potentially lead to unaddressed local research needs, failure of research to inform policy or unsustainable research capacity to respond to future outbreaks. The UKCDR and GloPID-R funders groups have further strengthened their response by agreeing to a set of Funder Principles for supporting high-quality research for the most pressing global needs in epidemics and pandemics ^5^ and with the formation of a new jointly hosted initiative for COVID-19 Research Coordination and Learning (COVID CIRCLE), encompassing the tracker and with a particular focus on resource-limited settings ^5^.

As part of the COVID CIRCLE initiative, this living mapping review has been established to regularly update and incorporate newly funded research projects as they become available and review their alignment to the WHO Roadmap priorities. A living mapping review (LMR) is needed due to the rapidly expanding number of funded research projects and the importance of the review to inform funding decision making. Here, in version three, we present the results of the second three-month update of all research projects within the tracker as of 15 ^th^ January 2020 and a descriptive and thematic analysis to aid interpretation of the global COVID-19 funded research portfolio. We have additionally published a more detailed analysis on the African continent specific baseline data from this tracker in collaboration with the African Academy of Sciences ^6^.

## Methods

### Protocol for LMR

The LMR protocol outlined herein was prospectively designed. Due to the rapid need for this project to be conducted to inform research responses during the pandemic, data extraction commenced before the protocol could be formally registered with PROSPERO. The protocol is outlined in this paper.

### Rationale for use of living method

Research funding bodies have responded rapidly to the COVID-19 pandemic through repurposing existing grants and rapidly funding projects with both rolling and one-off funding calls. This has resulted in new research projects being funded at short intervals necessitating a living review for this work. The regular update of this review will help coordinate ongoing researcher and funder responses.

### Eligibility criteria

All research projects funded by any research funder around the world (including regional funding organisations, national research funders and non-profit/ philanthropic organisations), with a focus on COVID-19 were eligible for inclusion in this analysis. This includes data from all types of research activities and was not limited to biomedical and health research. Furthermore, this analysis includes grants identified by funders as having been repurposed to address COVID-19 research priorities.

### Information sources and search strategy

The database and subsequent analysis make use of data from publicly-announced COVID-19 research grants and were obtained using one of two methods. Data was either obtained through direct communication with research funders by requesting the completion of a template spreadsheet (Extended data 1 ^7^). These requests were made to UKCDR and GloPID-R funder groups members ^8, 9^ on a regular basis (as part of funder coordination meetings) and to wider funder contacts beyond these groups. Alternatively, data were also obtained from online databases belonging to research funders using “COVID” and/or “coronavirus” as search terms (see Extended Data 2 ^7^). The tracker remains open to the submission of new funding data relating to COVID-19 from any global funder at any time. Screening of submitted data occurs on a weekly basis.

As the database is updated, a regular review is conducted to identify duplicate entries. Where duplicates are removed from the tracker the entry with the most detailed information is retained.

Though the set of data fields varied between funders, the data fields presented in Table 1 were considered a priority for the purposes of the tracker and subsequent analyses:

**Table 1.** Priority data fields for the UK Collaborative on Development Research (UKCDR) and Global Research Collaboration for Infectious Disease Preparedness (GloPID-R) tracker and analysis. The latest and previous versions of this table are available as extended data ^7^.

Data Field	Definition
**Abstract**	Scientific summary of the project
**Amount awarded**	Total amount awarded by the responsible funder for the duration of the project (with currency stated)
**Country(-ies) where studies are** **being conducted**	All countries where research is being conducted
**Funder(s)**	The names of all funding organisations (including co-funding)
**Lead institution**	The name of the organisation that holds the grant and is leading the research
**Local implementing partner(s)**	The name of any partner institutions located in the country(-ies) where the study is being conducted
**Principal investigator**	Name of the awarded project’s lead investigator based at the lead institution (primarily used for project de-duplication)
**Project ID/reference number**	Any unique reference number / project ID assigned by the funder organisation to this project (primarily used for project de-duplication)
**Project title**	Title of the research project
**Start/end date**	Start and end dates of the project

### Update schedule

All figures will be updated on a three-monthly basis; the discussion will also be revised to reflect any changes and trends over time. This living review will continue to be updated for the duration of the COVID CIRCLE initiative funding. The frequency of screening will not be reduced for the duration of COVID CIRCLE, although updates will only continue where new grants are included.

### Manually coded data fields

Data entry of additional manually classified variables was completed by one reviewer with each variable cross-checked by a second reviewer. Abstracts in French, German, Portuguese and Spanish were coded by project team members fluent in those languages or translated into English using Google Translate. Projects were coded against the following classifications:

*1. WHO medium-long term research priorities and sub-priorities*

Projects were assigned to one or more WHO priority areas of primary focus (Extended data 3 ^7^). An assignment of ‘N/A’ was made where: information provided was insufficient for classification; funds were allocated for research administration; or where projects clearly fell outside the WHO broad priority areas. Subsequently, projects were assigned to appropriate WHO sub-priority area(s). The assignment of ‘N/A’ was made if insufficient information limited further sub-categorisation or the projects fell outside the WHO sub-priority areas. In addition, suitable secondary priority area(s) with corresponding sub-priority(ies) were determined for those projects that significantly addressed other priority areas. Hence, projects were assigned with multiple primary and/or secondary WHO priority and sub-priority areas of research focus. The priority list will be updated if future iterations of the WHO Roadmap are released.

*2. Emergent categories for research falling outside the WHO priority classification*

For those projects that were not considered as addressing any of the WHO Research Priorities, they were assigned ‘N/A’ and new sub-priorities were developed and assigned on an initial data set of 400 projects. An inductive approach was used to develop new codes that emerged from the funded research and themes were confirmed through an iterative process through the projects in the baseline assessment. Six new sub-priority codes were defined under the social science priority (mental health; digital health; policy and economics; education; logistics and food security). A new priority focusing on the environmental impacts of COVID-19, was developed as well. All newly identified categories were validated using the full baseline dataset. In version two one further new emergent category, long COVID, was identified within the clinical management priority, however this has now been reclassified as a cross-cutting theme (see below) due to research on long COVID now being funded across a range of research priority areas

*3. COVID-19 Research Priorities for LMICs*

Research projects involving LMICs were additionally assessed for their alignment with the research priorities identified in a collaborative study conducted by the UKCDR, African Academy of Sciences (AAS) and the Global Health Network (TGHN) in May 2020 ^10, 11^. This study, which determined globally relevant COVID-19 research priorities with a specific focus on less-resourced countries, was based on earlier work by the AAS to determine the COVID-19 research priorities for Africa ^10^ and the mid- to long-term research priorities summarized in the WHO Research Roadmap ^2^. The study findings, published in August 2020, outline existing WHO research priorities which require greater research emphasis and new research priority areas not captured in the WHO Roadmap or identified in the AAS survey (Extended data 4 ^7^). Each funded research project involving LMICs was assigned to one of the new categories outlined in Supplementary material 2 or noted if it fell outside both the new AAS priorities and the new priorities identified by the UKCDR/AAS/TGHN study.

*4. Cross-cutting themes*

During the data coding process, a number of cross-cutting themes identified by the project team were coded for analysis. For this version three, the following additional variables were identified (classified as yes or no): capacity strengthening; cohorts; gender; implementation; indirect health impacts; innovation; modelling; pandemic preparedness; repurposed projects; and long COVID.

*5. Study population*

A study population categorisation structure was proposed using an inductive approach on an initial data set of 400 projects and validated using the full data set, allowing the categories to be specific to the populations represented in the funded research.

For the purposes of this analysis, a hierarchical categorisation system was produced to examine the study populations of the research projects included in the tracker. At the highest levels, research projects are assessed on whether they involve animal populations, human populations, literature reviews, policy analysis or only focus on the virus itself. Research projects focused on human populations, were classified against three additional sub-categories. Table 2 outlines the categories, sub-categories and levels in full.

**Table 2.** Study population categorisation system. The latest and previous versions of this table are available as extended data ^7^.

Level	Category	Sub-category
**1**	**Population**	Animal population
		Human population
		Literature reviews
		Policy
		Virus
		Other
**2**	**Human sub-population**	Adults
		Adults- women
		Adolescents
		Children
**3a**	**Population group- vulnerable** **populations**	Care home patients
		Disabled
		Domestic Violence Victims
		Elderly
		High risk individuals (defined as such in the study)
		LGBTQI+ community
		Minority communities (defined as such in the study)
		Neonates
		Pregnant women
		Refugees
**3b**	**Population group- Frontline** **workers - Healthcare workers**	Care home staff
		Doctors
		Informal
		Nurses
		Paramedics
		Social care workers
**3c**	**Population group- Frontline** **workers - Non-healthcare**	Firefighters
		Sanitation
		Volunteers
**4**	**COVID-19 infection status**	Negative
		Negative – Recovered
		Positive
		Positive – Severe

LGBTQI+ - Lesbian, gay, bisexual, transgender, queer and intersex, COVID-19 – Coronavirus disease 2019.

### Synthesis of results

Descriptive and comparative analyses are used in this analysis to present a description of funded COVID-19 research included in the tracker database as of 15 ^th^ January 2020.

The data used for this analysis can be obtained from the COVID-19 Research Project Tracker page on UKCDR’s website, as mentioned in the data availability statement. Data on the tracker (and subsequent analyses) will continue to be updated as more data becomes available and are obtained by the project team.

The charts and figures produced in this analysis were produced using Microsoft Office (Office 365 versions of Excel and PowerPoint) and (version 2020.2)

### Limitations of the data

Among the main challenges of the analysis is the varying completeness of data which led to less refined categorisation (assignment of projects to broad priority but notsub-priority areas) where the qualitative details of projects provided were insufficient. Therefore, the assigned priority areas may have failed to capture all aspects of the projects relevant to the WHO Roadmap. The same can be said for any value that was assigned to a given research project by the project team, including the study population and type of research activity. The data validation process by reviewers with expertise in global health research, policy, and funding outlined in the Project Selection section was used to address this and ensure that any assigned value was as accurate as possible, given the information provided.

Data on funding amounts was available from 62 of 101 funders (70.2% of all projects) and as a result this analysis is limited in providing a full financial profile of COVID-19 research funding investments. However, as the analysis makes use of all publicly available information it can therefore, be considered the most comprehensive characterisation possible.

At a higher level, the comprehensiveness of the tracker is limited to the funders that have either provided data for the tracker or had their data extracted from online sources (if available). In this respect, there were challenges in engaging with (and obtaining data from) health research funders beyond existing networks either due to a lack of contacts or capacity from funders to contribute to the project (especially for funders whose award information is not in English). Few funders have yet identified or made available details on grants repurposed towards COVID-19 to date although we continue to request this

### Risk of bias

This LMR of funded COVID-19 research projects uses descriptive and thematic analysis to summarise the scope of funded COVID-19 research projects. No attempts are made to assess the quality of individual studies or whether the studies meet their objectives. The potential sources of bias with project selection, quality of data reviewed, and data extraction and classification are addressed by robust fortnightly searches, template completion by funders and independent assessment and review during project classification respectively, as mentioned in the Information Sources and Search Strategy.

While the intention of the tracker and subsequent analyses are to provide as comprehensive a picture as possible of the COVID-19 research landscape, the data obtained for the tracker is more likely to be derived from funders of research that are members of UKCDR (all UK and broad disciplinary focus) and/or GloPID-R (global membership spanning HICs to LICs with a majority of national funders, and a biomedical focus). This would likely skew the results to show that more research being funded from these organisations and reflect trends in their respective portfolios (in terms of location, research focus and research activity type) than may necessarily be the case of the landscape more generally. In particular, 93.4% of the research projects included in the latest version of the tracker are from funders based in 27 high-income countries.

## Results

### Project selection

**Figure 1.**
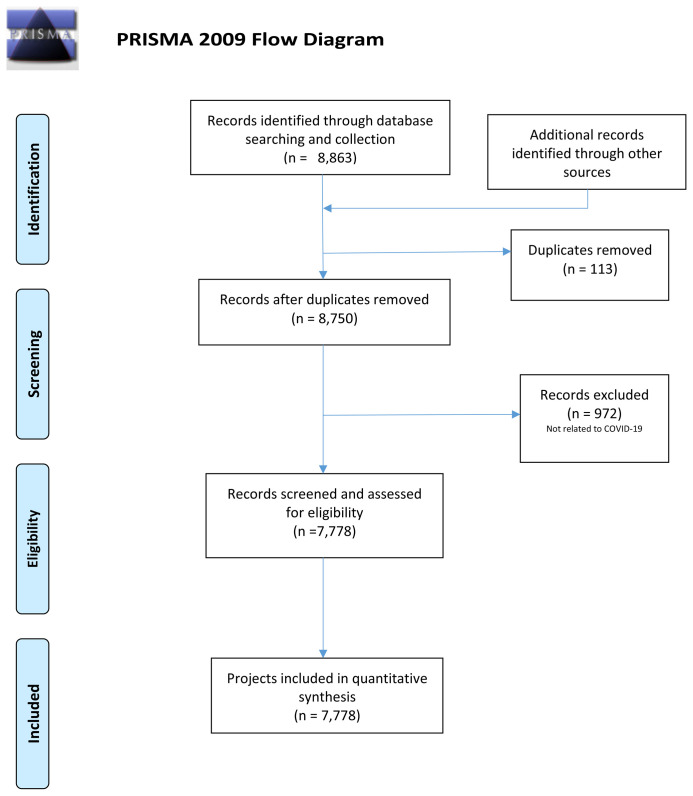
PRISMA flow diagram. The latest and previous versions of this figure are available as extended data ^7^ .

In total, 8,863 projects were assessed against the eligibility criteria outlined in the methodology and 1,085 were excluded for being duplicate projects or failing to meet the eligibility criteria as they were not related to COVID-19 (PRISMA Flow Diagram provided in Figure 1). The remaining 7,778 projects were assigned to the manually-coded data fields by nine project team members before being validated by an independent reviewer not involved with the initial screening and assigning process. This represents an increase of 2,694 projects as a result of the update to the analysis. All reviewers have broad expertise in global health research, policy, and funding.

### Project characteristics

Summaries of the characteristics of the 7,778 projects included in the latest version of the tracker are provided in the discussion of the results (below) which breaks down the projects by:

- Funder;
- Priority and sub-priority areas;
- Location;
- Activity type;
- Study population.

A full list of the projects is provided as underlying data ^12^.

**Figure 2.**
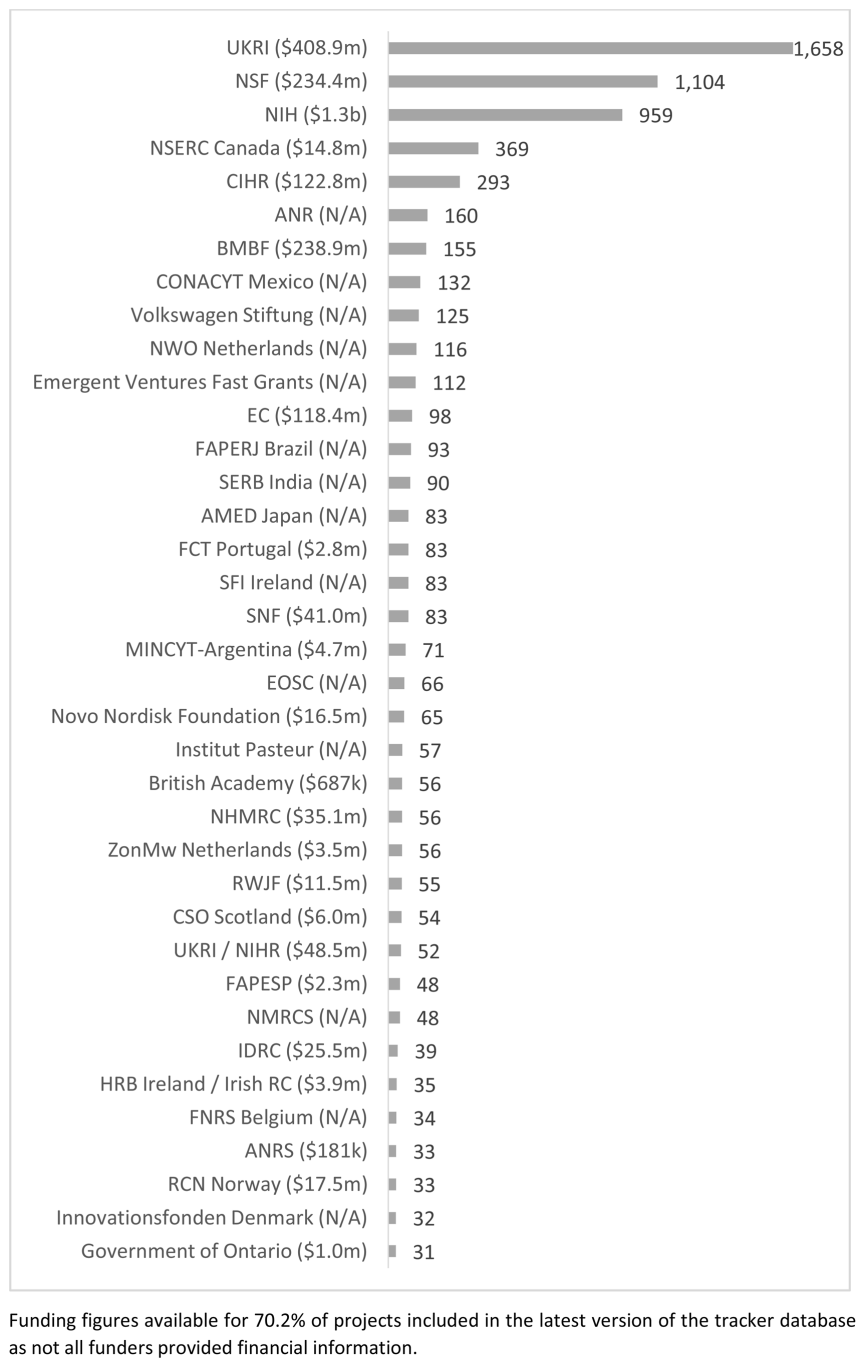
Number of Projects by Research Funder (funders with at least 30 projects on the latest version of the tracker displayed).

***Project funder.*** The 7,778 projects included in the latest version of the tracker comprises of data compiled from 100 research funders based in 35 different countries representing an investment of at least $3.8 billion (funding amounts only available for 70.2% of projects) ( Figure 2). This represents a more than 40% increase in the number of funders (previously 71 funders based in 28 countries) since the previous version of the analysis.

Known funding amounts indicated in brackets*. The latest and previous versions of this figure are available as extended data ^7^. **Funders with less than 30 projects: Medical Research Future Fund (Australian Government)** (29 projects; $46.8m); **NIHR** (28 projects; $22.0m); **National council for Scientific Research Lebanon** (28 projects; N/A); **Swedish Research Council** (27 projects; $8.2m); **Saskatchewan Health Research Foundation** (27 projects; $170k); **Royal Academy of Engineering** (25 projects; $649k); **Department of Science, Technology and Innovation, Colombia** (25 projects; $7.0m); **Michael Smith Foundation** (24 projects; N/A); **Health Research Council New Zealand** (24 projects; $6.8m); **Formas** (23 projects; $3.5m); **Academy of Finland** (22 projects; $21.8m); **REACTing/ INSERM** (20 projects; N/A); **Sino-German Center** (20 projects; N/A); **FWO Belgium** (20 projects; $5.6m); **EDCTP** (20 projects; $7.7m); **Ministry of Higher Education and Science, Denmark** (19 projects; $14.1m); **National Science Center Poland** (19 projects; $3.3m); **Lundbeck Foundation** (18 projects; $4.6m); **Carlos III Health Institute** (18 projects; $5.6m); **APPRISE** (18 projects; $498k); **DFID, NIHR, Wellcome (Elrha funding call)** (15 projects; $1.4m); **Danish Independent Research Foundation** (15 projects; $3.5m); **Austria Science Fund** (14 projects; $4.0m); **Wellcome / DFID** (13 projects; $17.3m); Research Manitoba (13 projects; $1.7m); **Estonian Research Council** (13 projects; $2.5m); **Roche** (11 projects; N/A); **Paul Ramsay Foundation** (via APPRISE) (11 projects; N/A); **Federal Office of Public Health of the Swiss Confederation** (11 projects; $3.7m); **Croatian Science Foundation** (11 projects; N/A); **German Research Foundation** (11 projects; N/A); **Coalition for Epidemic Preparedness Innovations** (11 projects; $864.5m); **Israel Innovation Authority** (10 projects; N/A); **AXA** (10 projects; N/A); **Wellcome** (9 projects; $1.8m); **NordForsk** (9 projects; N/A); **Peter Wall Institute** (8 projects; N/A); **Innovations for Poverty Action/ DFID** (8 projects; N/A); **ANR / Other Funders (France)** (8 projects; N/A); **Victoria State Government (Australia)** (7 projects; N/A); **New South Wales Government** (7 projects; N/A); **Innosuisse** (7 projects; $1.7m); **DIM-ELICIT** (7 projects; N/A); **APPRISE/CREID** (7 projects; N/A); **Alberta Innovates** (7 projects; $1.6m); **RCN / Trond-Mohn Foundation** (6 projects; $3.1m); **Wellcome Centre for Infectious Diseases Research in Africa** (6 projects; $338k); **SFI Ireland / IDA Ireland / Enterprise Ireland** (5 projects; $2.0m); **Carlsberg Foundation** (5 projects; $6.0m); **Snow Medical (via CREID / APPRISE)** (4 projects; N/A); **NRF (Korea)/ Swedish Research Council** (4 projects; $720k); **Nordic Trial Alliance** (4 projects; N/A); **CREID** (4 projects; N/A); **NSFC/NRF (Korea)** (3 projects; N/A); **Fondation pour la Recherche Médicale** (3 projects; $579k); **Banque publique d'investissement France** (3 projects; $21.8m); **NSFC/BNSF** (2 projects; N/A); **COVID-19 Therapeutics Accelerator (Wellcome / Bill & Melinda Gates Foundation)** (2 projects; $11.0m); **Wellcome and co-funders** (1 project; N/A); **UNITAID / EDCTP** (1 project; N/A); **UNITAID / ANRS** (1 project; N/A); **Federal Food Safety and Veterinary Office, Switzerland** (1 project; 138k); **Department for Environment, Food and Rural Affairs, UK** (1 project; 30k); **Chan Zuckerberg Initiative** (1 project; N/A); **Other Funders (Canada)** (260 projects; N/A); **Other funders (France)** (37 projects; N/A). **Abbreviations and Acronyms: AMED**- Agency for Medical Research and Development; **ANR - **Agence nationale de la recherche (National Research Agency); **ANRS** - Agence nationale de recherche sur le sida et les hépatites virale (National Agency for AIDS Research); **APPRISE** - Australian Partnership for Preparedness Research on Infectious Disease Emergencies; **BMBF** - Bundesministerium für Bildung und Forschung (German Federal Ministry of Education and Research); **BNSF** - Bulgarian National Science Fund; **CIHR** - Canadian Institutes of Health Research; **CONACYT** - Consejo Nacional de Ciencia y Tecnología (Mexico National Council of Science and Technology); **CREID** - Centre of Research Excellence in Emerging Infectious Diseases; **CSO** - Chief Scientist's Office; **DFID** - Department for International Development; **EC** – European Commission; **EDCTP** - European & Developing Countries Clinical Trials Partnership; **EOSC** - European Open Science Cloud; **FAPERJ** - Fundação de Amparo à Pesquisa do Estado do Rio de Janeiro (Research Foundation of the State of Rio de Janeiro); **FAPESP** - Fundação de Amparo à Pesquisa do Estado de São Paulo (São Paulo Research Foundation); **FCT** - Fundação para a Ciência e Tecnologia (foundation for Science and Technology); **FNRS** - Fonds de la Recherche Scientifique (National Fund for Scientific Research); **FWO** - Fonds voor Wetenschappelijk Onderzoek – Vlaanderen (Research Foundation Flanders); HRB - Health Research Board; **IDRC** – International Development Research Centre; **INSERM** - Institut national de la santé et de la recherche médicale (National Institute of Health and Medical Research); **Irish RC** – Irish Research Council; **MINCYT** - Ministerio de Ciencia, Tecnología e Innovación (Argentina Ministry of Science, Technology and Innovation); **NIH** - National Institutes of Health; **NIHR** - National Institute for Health Research; **NHMRC** - National Health and Medical Research Council; **NMRCS** - National Medical Research Council Singapore; **NRF (Korea)** - National Research Foundation of Korea; **NSERC** - Natural Sciences and Engineering Research Council; **NSF** - National Science Foundation; **NSFC** - National Natural Science Foundation of China; **NWO** - Nederlandse Organisatie voor Wetenschappelijk Onderzoek (Dutch Research Council); **RCN** - Research Council of Norway; **REACTing** - REsearch and ACTion Targeting Emerging Infectious Diseases; **RWJF** - Robert Wood Johnson Foundation; **SERB** - Science and Engineering Research Board; **SFI** - Science Foundation Ireland; **SNF** - Schweizerischer Nationalfonds zur Förderung der wissenschaftlichen Forschung (Swiss National Science Foundation); **UKRI** - UK Research and Innovation; **ZonMw** - Nederlandse organisatie voor gezondheidsonderzoek en zorginnovatie (Netherlands Organisation for Health Research and Development).

With the updated data, nearly one third of funded projects in the tracker were awarded by funders based in the United States (28.8%) – ranking first among all other countries, ahead of the United Kingdom (24.7%) and Canada (13.8%). In terms of known funding amounts, funders based in the United States were again ranked first among all other countries, collectively investing $1.6b (41.6% of available financial data). However, data from the additional 2,694 projects has resulted in the $885m invested by Norwegian-based funders (notably including the Coalition for Epidemic Preparedness Innovations (CEPI)) ranking second among all other countries (23.4% of total known funding amount) ahead of the $518.m invested by United Kingdom-based funders (13.7% - previously second).

***Categorisation of projects against WHO Roadmap priorities & sub-priorities.*** All projects were categorised against the priorities and sub-priorities identified by the WHO in their Coordinated Global Research Roadmap, with several research projects being assigned multiple priority and/or sub-priority areas.

**Figure 3.**
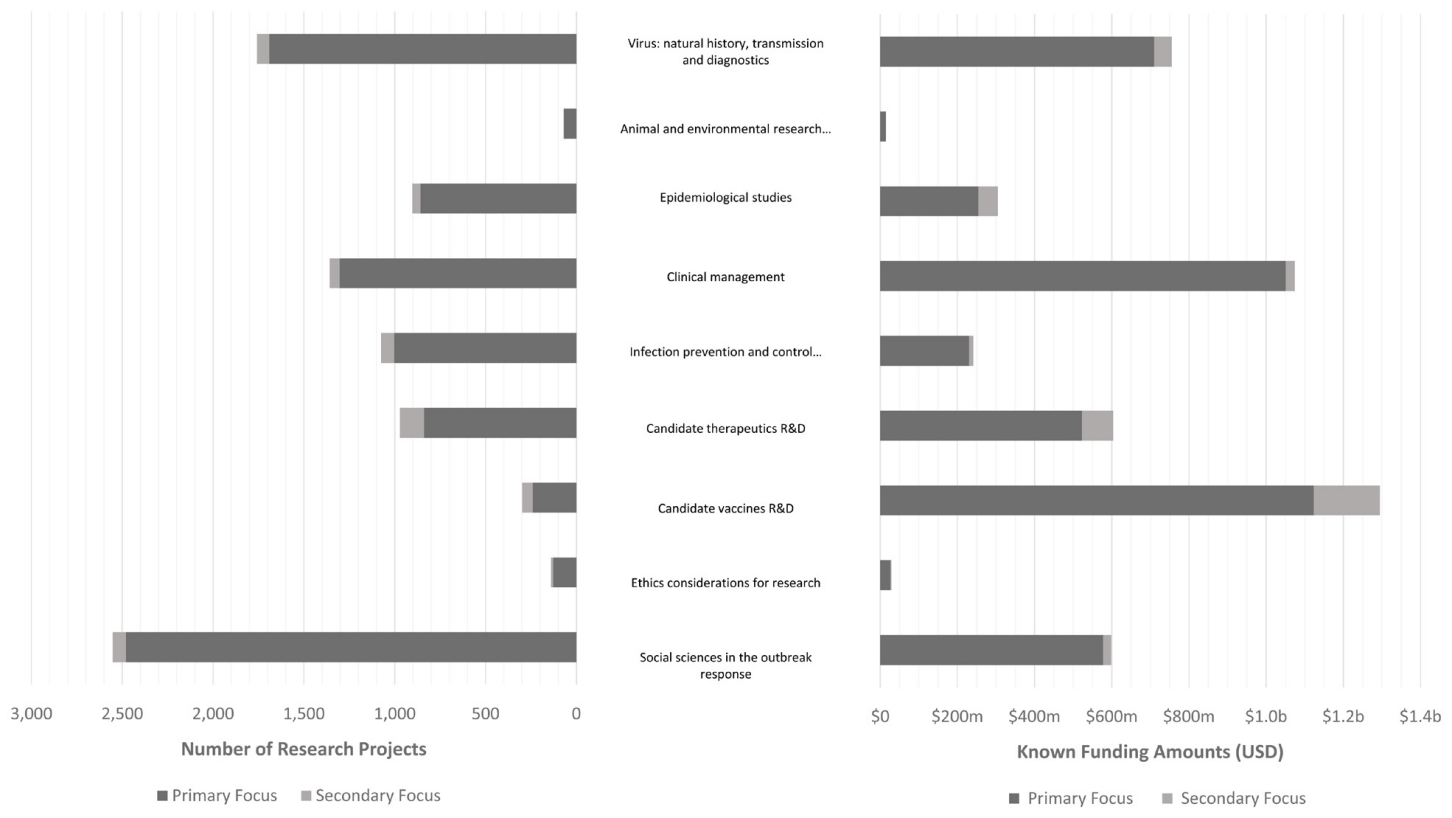
COVID-19 Research Projects Classified Against Priorities Outlined in WHO Coordinated Global Research Roadmap. The latest and previous versions of this figure are available as extended data ^7^.

***WHO Priority Areas.*** Figure 3 displays both the number of projects listed under each priority area and the known funding amounts (as not all funders provided financial information for their awarded research projects). The priority area under ‘Candidate Vaccines R&D’ now ranks first among all nine priority areas in terms of known funding amounts (previously second) at $1.3b (increasing by $821.5m or 173.7% since to the second version of the analysis).

However, while ranking first in terms of known funding amounts, the ‘Candidate Vaccines R&D’ priority area ranks seventh in terms of the total number of research projects (298) highlighting the different grant values for different types of research funded in response to the COVID-19 pandemic. The average value of ‘Candidate Vaccines R&D’ projects was $4.3m five-times the priority area with the second-largest average grant amount (‘Clinical Characterization and Management at $793k) and more than twenty-times the priority area with the smallest average, ‘Ethics Considerations for Research’ ($209k).

Following the inclusion of 2,694 additional projects to the tracker database, the two priority areas of ‘Animal and environmental research on the virus origin, and management measures at the human-animal interface’ (71 projects totalling $15.1m) and ‘Ethics considerations for research’ (139 projects totalling $29.0m) continue to receive relatively little funding from the fewest research funders (19 and 35, respectively). The cross-cutting nature of ethics considerations for research however means that much work on this will occur within other research projects.

**Figure 4.**
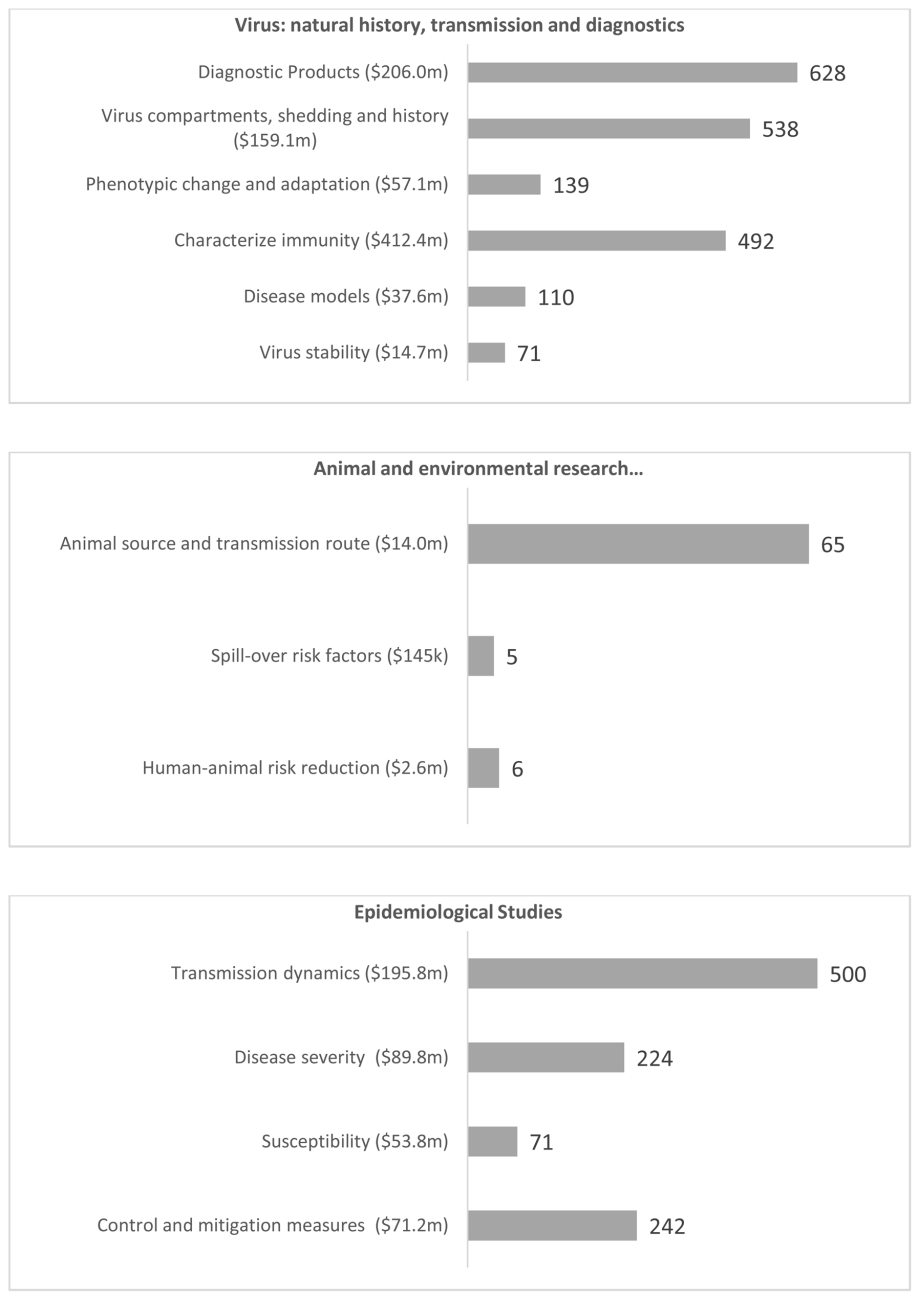
Number of Research Projects Included Under Each Sub-Priority Outlined in WHO Coordinated Global Research Roadmap (known funding amounts indicated in brackets). The latest and previous versions of this figure are available as extended data ^7^.

***WHO Sub-Priority Areas.*** Figure 4 shows how the 7,778 COVID-19 research projects included in the latest version of the tracker have been categorised against abbreviated versions of the 44 sub-priorities mentioned in the WHO Roadmap. The names of the sub-priorities are listed in full as Extended data ^7^.

The funding patterns at the WHO priority-level are reflected at the sub-priority level. Notably, seven of the nine sub-priorities with the greatest number of research projects are from the three priority areas with the greatest number of projects – namely ‘Virus: natural history, transmission and diagnostics’ (3 sub-priorities), ‘Social sciences in the outbreak response’ (2) and ‘Clinical Characterization and Management’ (2).

Similarly, seven of the ten sub-priorities with the greatest investment totals are from priority areas ranked among the top four for funding amounts – namely ‘Virus: natural history, transmission and diagnostics’ (again with 3 sub-priorities), ‘Clinical Characterization and Management’ (2) and ‘Candidate Therapeutics R&D’ (2). Additionally, the top two sub-priority areas with the largest average amount of known funding are both from the ‘Candidate Vaccines R&D’ priority area.

More specific research investment gaps emerge within the priority area with the lowest level of investment. While the priority area ‘Animal and environmental research on the virus origin, and management measures at the human-animal interface’ consists of just three sub-priority areas, the ones focussing on 'Socioeconomic and behavioural risk factors for spill-over' (5 projects worth $145k) and 'Risk reduction strategies at the human-animal environment interface' (6 projects worth $2.6m) rank very low among all 44 sub-priorities in terms of both number of projects (joint-last and 41 ^st^, respectively) and known funding amounts (last and 39 ^th^, respectively). The other notable sub-priority with limited attention relates to ‘Develop core clinical outcomes to maximise usability of data across range of trial’ under the ‘Clinical characterization and management’ priority area which is the focus of 5 projects (ranked joint-last) worth $1.8m (ranked second-to-last).

**Figure 5.**
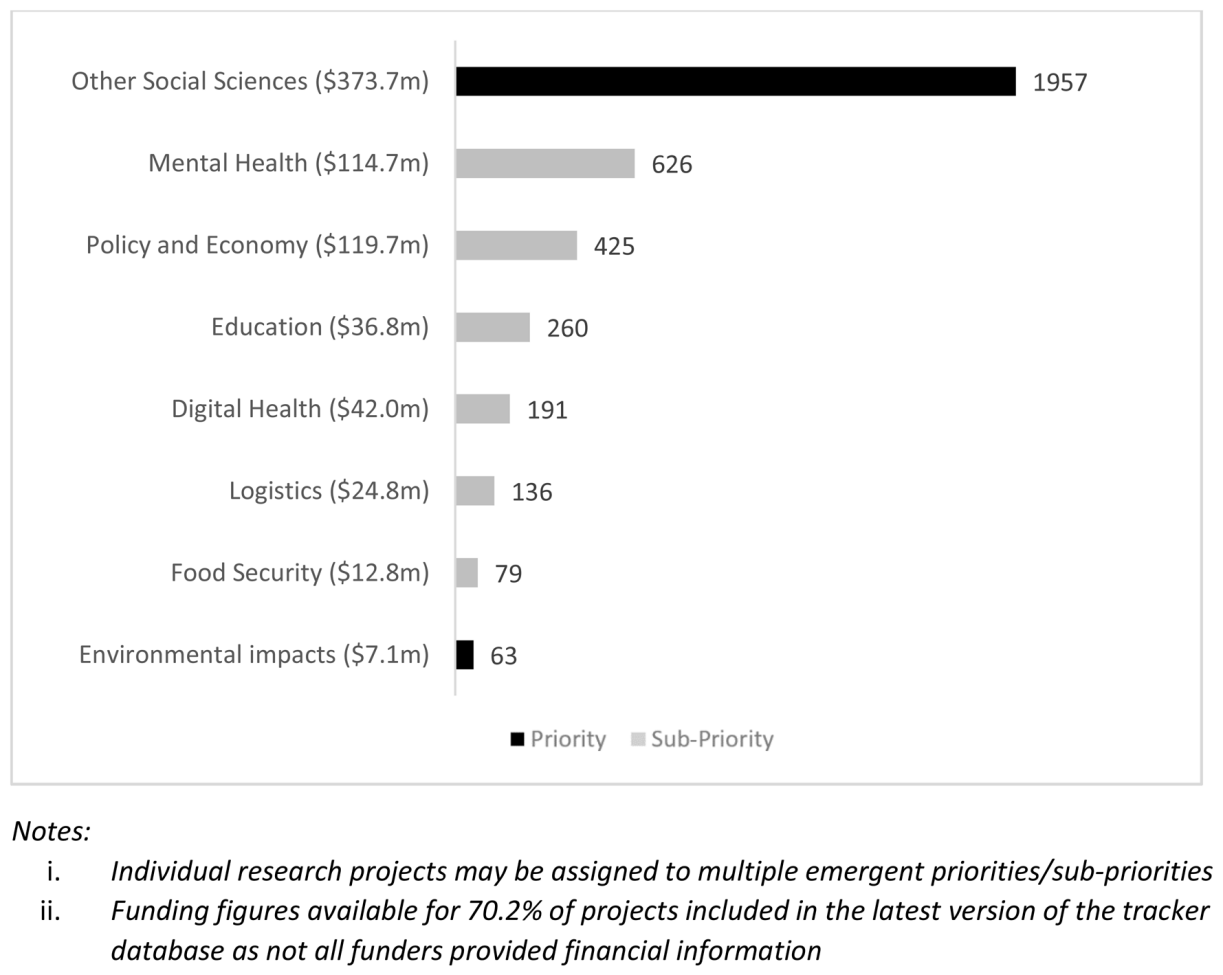
Number of research projects classified against emergent research priority and sub-priority areas not previously identified (known funding amounts indicated in brackets). The latest and previous versions of this figure are available as extended data ^7^.

***Classification of research projects which did not categorise against WHO Roadmap (emergent categories).*** The majority of research projects which did not categorise against the WHO Roadmap are social sciences research that did not align with the sub-priorities in the WHO Roadmap. These are shown by the newly created categories in Figure 5. These highlight important emergent themes for COVID-19 research prioritised by, both researchers and funders.

‘Mental health’ is the most prominent emergent category which does clearly fall within a health remit and it is notable both that this type of research is receiving much attention, but also that currently this is limited to research from a social science perspective, rather than a clinical perspective (and the same can also be said of research addressing ‘digital health’). The further emergent social sciences related sub-priorities of ‘policy and economy’, ‘education’, ‘logistics’ and ‘food security’ and emergent priority of ‘environmental impacts’ are all focussed on the broader social and economic impacts of the COVID-19 recovery and reflect the broader COVID-19 research focus of the tracker and the research consideration of the knock-on effects (health and otherwise).

**Figure 6.**
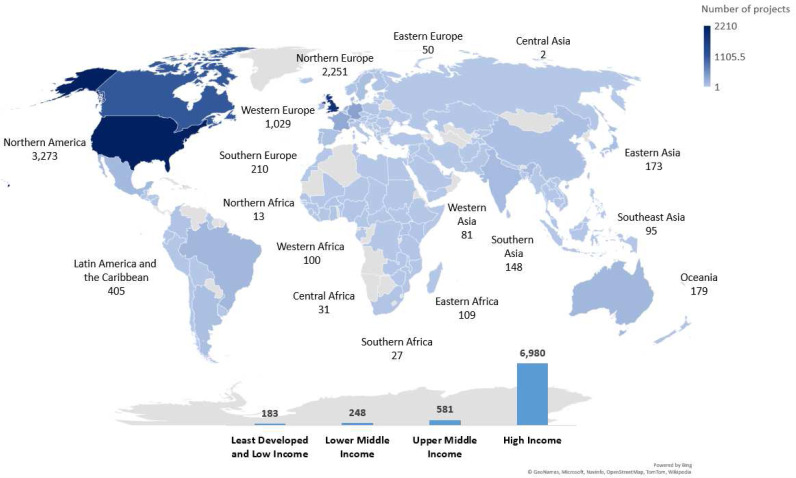
Location of coronavirus disease 2019 (COVID-19) research projects by country, WHO sub-region and Organisation for Economic Co-operation and Development’s (OECD) Development Assistance Committee (DAC) list categories. The latest and previous versions of this figure are available as extended data ^7^.

***Location of projects.*** Figure 6 summarises the location where research projects are taking place. Research is being conducted in 136 countries with the greatest number of projects taking place in the United States (2,210 projects) followed by the United Kingdom (1,847) and Canada (1,073).

Of the 7,778 research projects, 325 (4.2%) take place across multiple countries, with research partnerships between Italy and Spain being the most common (30 projects).

Classifying countries by income categories (using the Organisation for Economic Co-operation and Development’s Development Assistance Committee list), nearly all research projects (88.8%) are taking place, at least in part, in high-income countries. With the updated analysis,the proportion of research projects taking place in at least one of the 77 Official Development Assistance (ODA) recipient countries has increased slightly from 10.1% (513 projects) to 10.9% (847 projects). Of these 847 projects, more than two-thirds (67.3%) are taking place in at least one upper-middle-income country.

Just 869 projects (11.2%) are taking place exclusively in LMICs (previously 8.0%) – with Brazil being the country with the greatest number of projects among these (153), followed by Mexico (137) and India (104). This ordering of countries is largely a result of the present selection of data in the tracker from funders based in these LMICs. Though 73 projects on the latest version of the tracker are taking place in China, it is acknowledged that there is much more nationally funded research occurring for which data has not yet been obtained. Among the 869 projects taking place exclusively in LMICs, 8.9% is being conducted across multiple countries.

**Figure 7.**
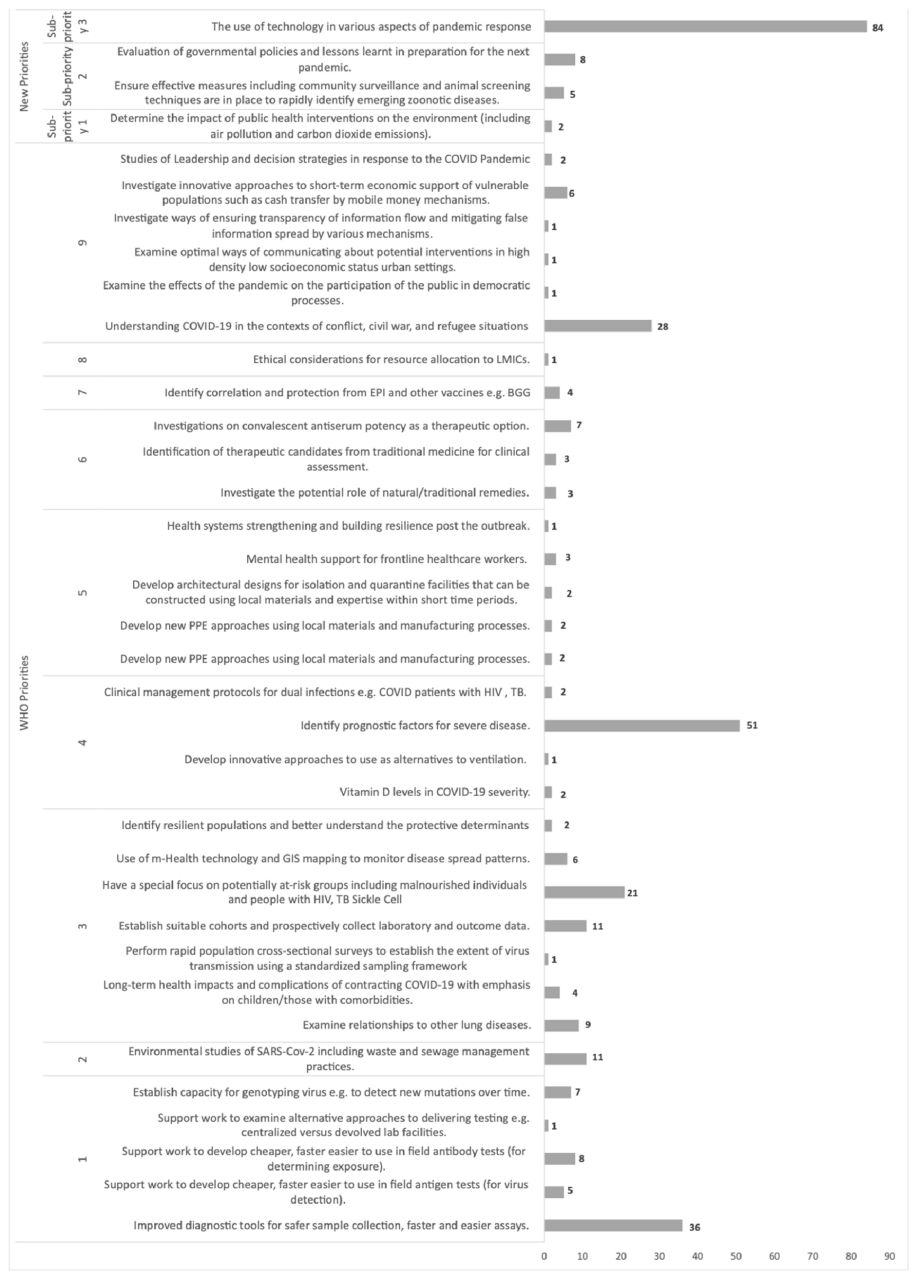
Research projects in LMICs categorised against their research priorities (LMIC Research Priorities). The latest and previous versions of this figure are available as extended data ^7^.

**Figure 8.**
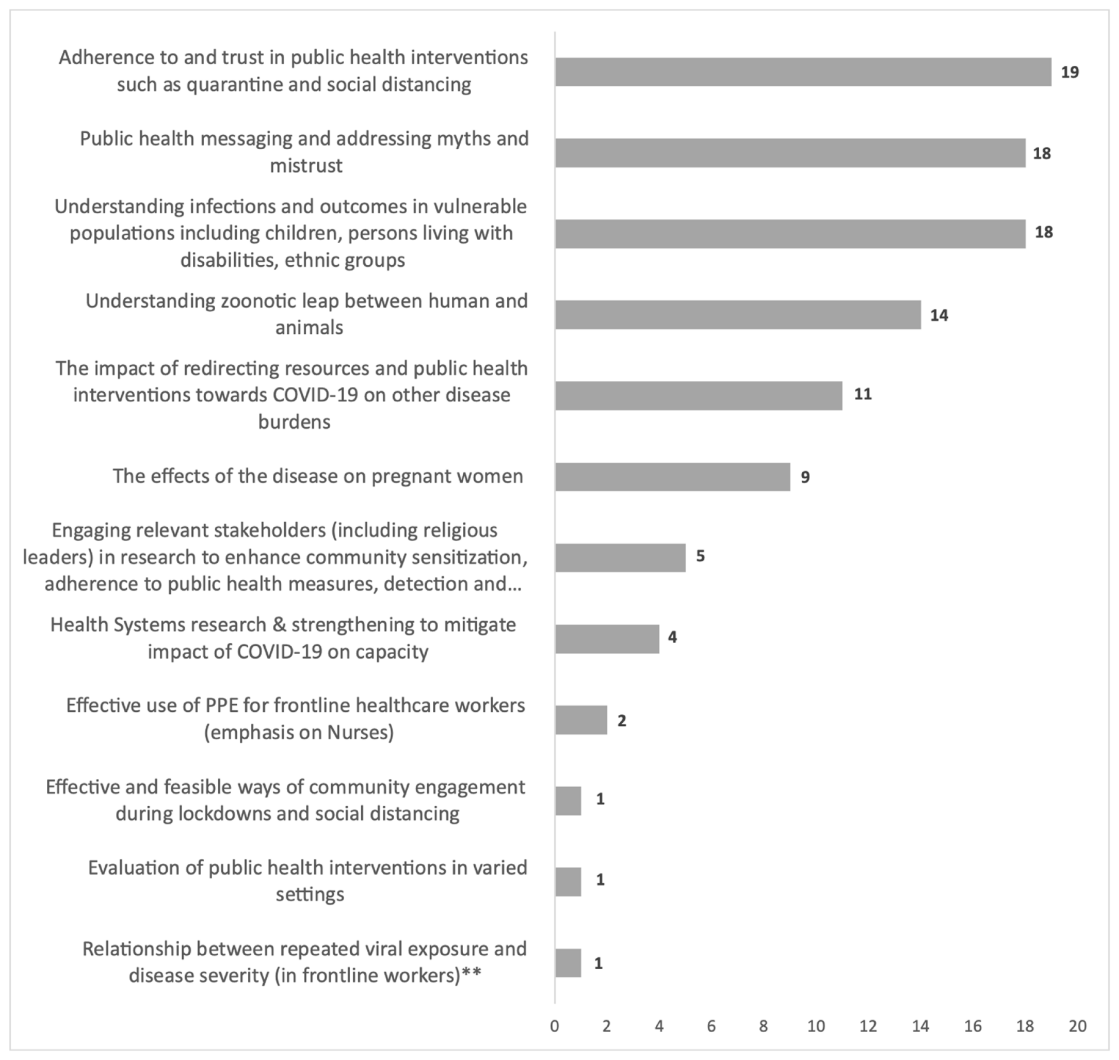
Research Projects in LMICs categorized against ‘existing WHO Priorities requiring greater research emphasis’. The latest and previous versions of this figure are available as extended data ^7^.

***Characteristics of research projects in Low- and Middle-Income Countries (ODA-recipient countries).*** Most research projects in low- and middle- income countries could be categorised against one or more WHO research priorities. In addition, several were also categorised against the context-specific research priorities identified by the UKCDR, African Academy of Sciences (AAS) and the Global Health Network (TGHN) and are shown in Figure 7 and Figure 8.

Figure 7 shows that some projects mapped to the context specific sub-priorities identified for LMICs under all the nine WHO priorities. The predominant theme was to ‘identify prognostic factors for severe disease’ whilst research to ‘develop improved diagnostic tools for safer sample collection, faster and easier assays’ ranked second. Similarly, a few projects mapped to the new broad priority areas with the highest category being the cross-cutting theme involving the use of technology in various aspects of the pandemic response. Figure 8 shows those projects mapping to existing WHO priorities ‘requiring greater research emphasis in LMICs’. Here most projects mapped to ‘ adherence to and trust in public health interventions’, ‘understanding infections and outcomes in vulnerable populations’ and ‘public health messaging and addressing myths and mistrust’ which might indicate the importance of these research areas in controlling the pandemic in LMICs. In contrast the highlighted priorities involving stakeholder engagement, health systems research, effective PPE use and examining the relationships between repeated viral exposure and diseases severity in frontline healthcare workers were lacking.

**Figure 9.**
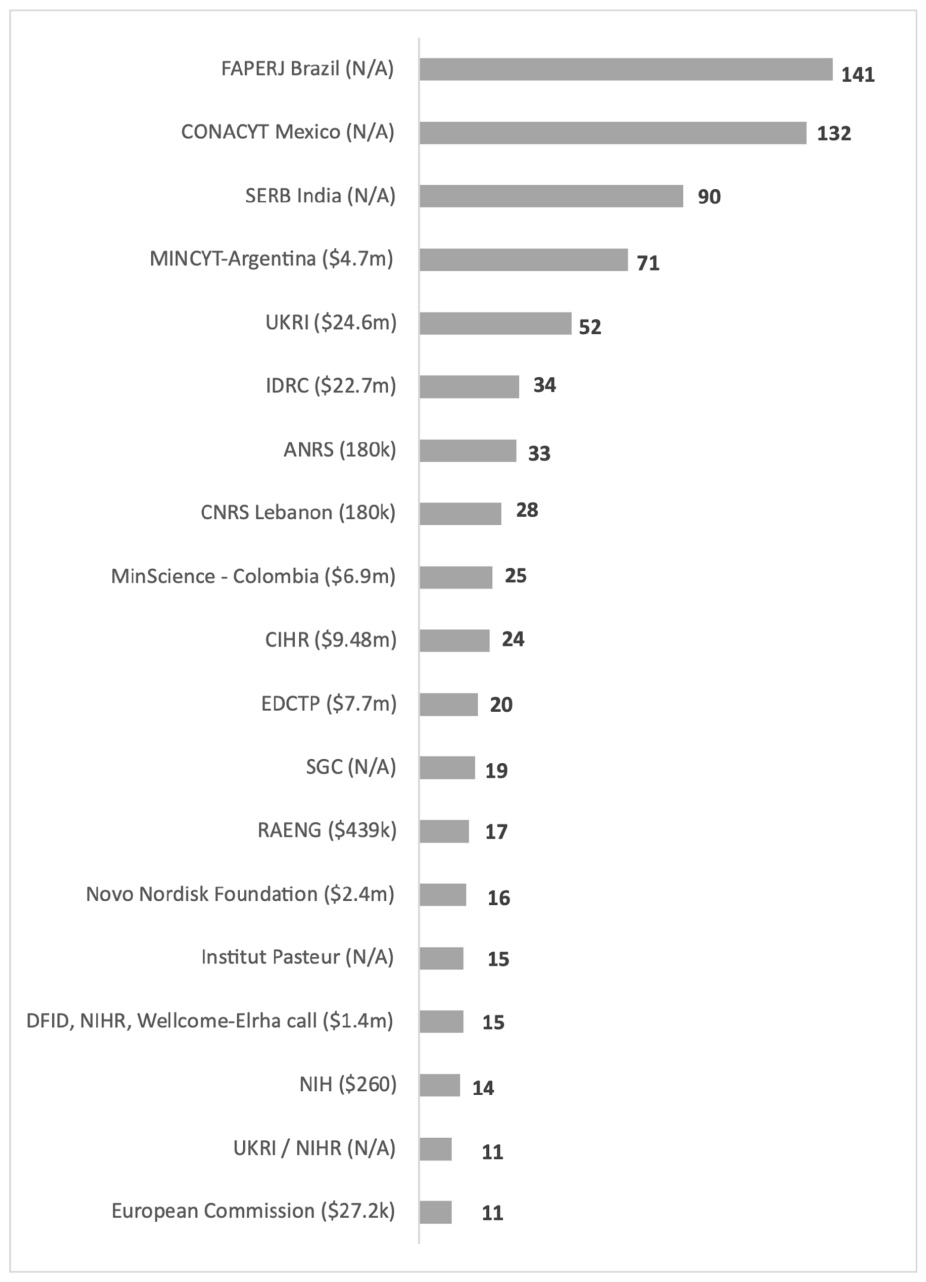
The Major Funders in ODA-Recipient Countries (funders with at least 10 projects on the latest version of the tracker displayed). Known funding amounts indicated in brackets*. The latest and previous versions of this figure are available as extended data ^7^. **Abbreviations and Acronyms: ANR -**Agence nationale de la recherche (National Research Agency); **ANRS** - Agence nationale de recherche sur le sida et les hépatites virale (National Agency for AIDS Research); **BMBF** - Bundesministerium für Bildung und Forschung (German Federal Ministry of Education and Research); **CIHR** - Canadian Institutes of Health Research; **CONACYT** - Consejo Nacional de Ciencia y Tecnología (Mexico National Council of Science and Technology); **DFID** - Department for International Development; **EC** – European Commission; **EDCTP** - European & Developing Countries Clinical Trials Partnership; **EOSC** - European Open Science Cloud; **FAPERJ** - Fundação de Amparo à Pesquisa do Estado do Rio de Janeiro (Research Foundation of the State of Rio de Janeiro); **FAPESP** - Fundação de Amparo à Pesquisa do Estado de São Paulo (São Paulo Research Foundation); **IDRC** – International Development Research Centre; **INSERM** - Institut national de la santé et de la recherche médicale (National Institute of Health and Medical Research); **MINCYT** - Ministerio de Ciencia, Tecnología e Innovación (Argentina Ministry of Science, Technology and Innovation); **NIH** - National Institutes of Health; **NIHR** - National Institute for Health Research; **NRF (Korea)** - National Research Foundation of Korea; **NSF** - National Science Foundation; **NSFC** - National Natural Science Foundation of China; **NWO** - Nederlandse Organisatie voor Wetenschappelijk Onderzoek (Dutch Research Council); **RCN** - Research Council of Norway; **REACTing** - REsearch and ACTion Targeting Emerging Infectious Diseases; **RWJF** - Robert Wood Johnson Foundation; **SERB**- Science and Engineering Research Board; **SFI** - Science Foundation Ireland; **SNF** - Schweizerischer Nationalfonds zur Förderung der wissenschaftlichen Forschung (Swiss National Science Foundation); **UKRI** - UK Research and Innovation.

Research Foundation of the State of Rio de Janeiro (FAPERJ), National Council of Science and Technology Mexico (CONACYT) and Science and Engineering Research Board (SERB) fund the most projects involving LMICs as shown in Figure 9. Of the funders not based in less-resourced countries UK Research and Innovation (UKRI), International Development Research Centre (IDRC) and ANRS (France REcherche Nord & sud Sida-hiv Hépatites - French Agency for Research on AIDS and Viral Hepatitis) fund the most research projects. Notably, these funders support research across multiple countries with UKRI-funded projects involving 41 different countries, most of which are concentrated in China and Uganda (where a UKRI MRC centre is located).

The National Science Foundation (NSF) has funded eight projects of which four involve China and three are described as being global. The majority of projects funded by Coalition for Epidemic Preparedness Innovations (CEPI) are in HICs with only 3 in China and India. This likely speaks to the availability of the requisite research capacity in HICs for carrying out preclinical and early stages of vaccine research which these projects are primarily concerned with.

***Cross-cutting themes.*** During the review and classification process projects were classified against ten additional characteristics: capacity strengthening; cohorts; gender; implementation; indirect health impacts; innovation; long COVID; modelling; pandemic preparedness and repurposed grants (descriptions of the types of projects classified against these are provided in the notes for Table 3). Table 3 summarises the distribution of the cross-cutting themes against the WHO Priority Areas.

**Table 3.** Number of Research Projects Included Under Each Cross-Cutting Theme and WHO Priority Area (known funding amounts indicated in brackets).

		WHO Priority Area									
Cross-cutting theme	Number of Projects	Virus: natural history...	Animal and env. research...	Epid. studies	Clinical char...	Infection prevention and control...	Candidate therapeutics R&D	Candidate vaccines R&D	Ethics...	Social sciences...	*N/A*
**Innovation** ($94.9m)	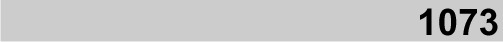	74	0	31	203	**321**	21	13	3	262	*198*
**Repurposed Grants ($342.8m)**	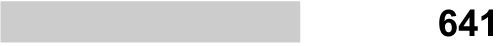	163	6	71	171	53	120	34	5	**222**	*49*
**Modelling** ($74.3m)	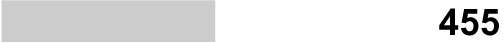	100	7	**241**	46	80	30	20	5	84	*12*
**Repurposed Grants** ($107.2m)	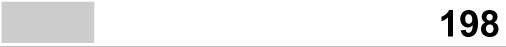	**56**	0	60	85	14	9	9	1	65	*6*
**Cohorts** ($239.1m)	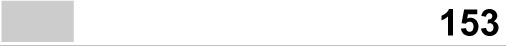	1	0	14	19	8	1	0	1	**126**	*12*
**Pandemic Preparedness** *($35.6m)*	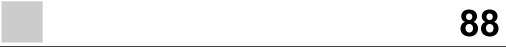	13	1	19	**73**	2	5	1	0	14	*1*
**Implementation** *($8.3m)*	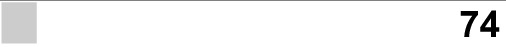	0	0	2	1	3	0	0	2	**72**	*1*
**Indirect Health Impact** *($6.9m)*	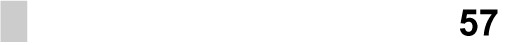	8	2	12	9	11	4	2	2	**35**	*3*
**Gender** *($4.2m)*	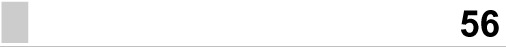	3	0	0	3	6	2	0	0	**13**	*34*
**Capacity Strengthening** **** *($9.3m)*	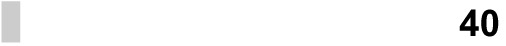	**10**	0	6	7	2	5	2	1	**13**	*6*

*Notes:* *i. Research projects may be assigned with multiple cross-cutting themes and WHO priority areas* *ii. Highlighted cells indicate the WHO Priority Area with the greatest number of projects for each cross-cutting theme (excluding projects that were not assigned a priority area, marked N/A).* *iii. Funding figures available for 69.9% of projects included in the latest version of the tracker database as not all funders provided financial information.* *iv. Definitions of cross-cutting themes-*     ◦ ***Capacity strengthening**: Projects which involve a capacity strengthening component. Capacity strengthening at all levels- individual, institutional and national is included.*     ◦ ***Cohorts**: Projects carried out in newly established cohorts or pivoted existing cohorts for COVID-19 research.*     ◦ ***Gender**: Projects which incorporate a gendered lens in description of methods/ objectives and project outputs.*     ◦ ***Implementation**: Grants for facilitating research administration.*     ◦ ***Indirect Health Impacts**: Projects focusing on indirect health impacts of COVID-19, for example related to disruptions in healthcare services, neonatal, maternal and child health, non-communicable diseases, chronic disease conditions and mental health.*     ◦ ***Innovation**: Projects involving novel inventions and interventions.*     ◦ ***Long COVID**: Projects involving the long-term morbidity and enduring symptoms of COVID-19 beyond the initial infection*     ◦ ***Modelling**: Projects involving any form of modelling in the methodology.*     ◦ ***Pandemic Preparedness**: Projects with preparedness for future pandemics as an objective.*     ◦ ***Repurposed projects:** Pre-COVID research grants (usually for MERS, SARS and other pathogens) where additional funding has been awarded for tailoring to COVID-19 research.*

Looking at the number of projects under each cross-cutting theme ( Table 3), the theme with the greatest number of projects, ‘innovation’, is largely driven by the large presence of UKRI data (766 projects accounting for 71.4% of these projects).

The vast majority of repurposed grants (82.7%) were funded by the National Institutes of Health (NIH) totalling $289.0m. The number of repurposed grants in the tracker is expected to increase as funders continue to make this data available (and has increased by $197.5m-worth of repurposed projects since the update to the analysis).

For the cross-cutting theme of ‘capacity strengthening’, more than half of these projects (62.5%) are taking place in at least one LMICs and, among these projects, mostly within the priority area of ‘Social Sciences in the Outbreak Response’ (13 of 40 projects taking place in at least one LMIC).

**Figure 10.**
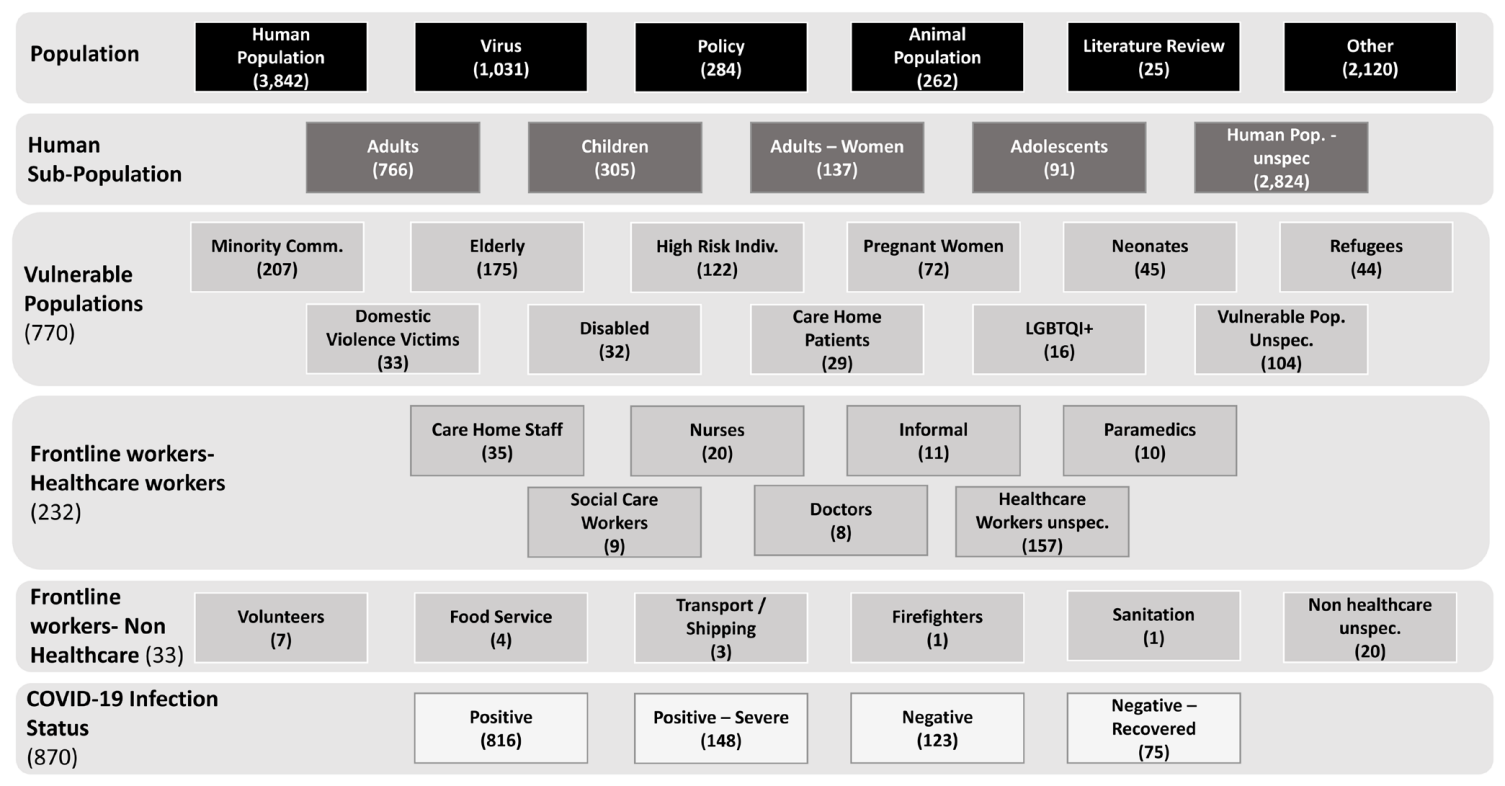
COVID-19 Research Projects Classified Using Study Population Categorisation System (number of projects indicated in brackets). The latest and previous versions of this figure are available as extended data ^7^.

***Study populations included in projects.*** Just under half of the research projects included in the latest version of the tracker deal with human populations (49.4%) with a significant emphasis on populations that have tested positive for COVID-19 (21.2% of research projects studying human populations) as well as population groups defined as vulnerable within the project (20.0% of research projects studying human populations). Figure 10 summarises how the research projects are classified across all levels of the study population categorisation system outlined above.

**Figure 11.**
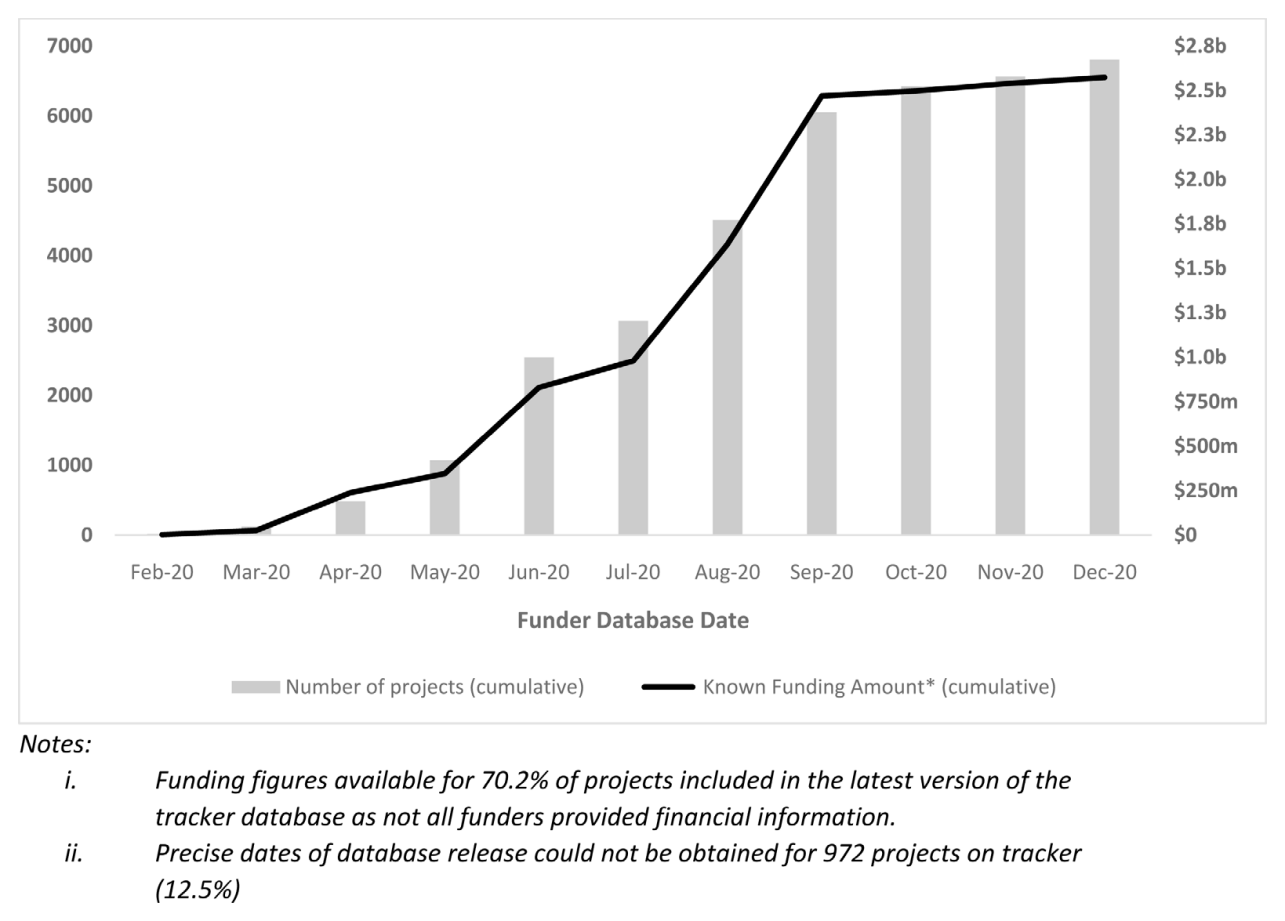
Cumulative number of projects and known funding amounts* by month of funder database release.

***Trends in Funding Over Time.*** To understand the progression of funding for COVID-19 research over time, Figure 11 summarises the cumulative sum of projects and funding amounts on the tracker according to the publication date of award information by funders (as opposed to the date when the details of projects were added to the tracker as there may be a lag between the time that funders publish award information and the time that such information is located and subsequently added to the tracker).

Of the additional 2,694 projects that were added to the tracker since the 15 ^th^ October 2020, only 25.7% came from funder databases that were known to be published between October and December 2020 and are therefore ‘newly funded or released’, while 41.6% came from databases that were known to be published prior to October and are therefore ‘newly captured’, but not newly funded (with the remainder not showing dates of release). More widely, Figure 11 shows a rapid expansion of projects funded over the summer of 2020 – particularly during August and September, with a plateauing of new funding from October 2020 onwards.

63.0% of projects taking place in at least one LMIC came from funder databases published between August and October 2020. These projects were largely funded by LMIC-based funders – most notably the National Council of Science and Technology of Mexico (132 projects), the Research Foundation of the State of Rio de Janeiro (93), and the Science and Engineering Research Board of India (90).

## Discussion

This six-month update review of the UKCDR and GloPID-R COVID-19 Research Project Tracker has described the huge investment and wide range of research projects repurposed or newly funded related to COVID-19 captured in the tracker between January 1 2020 and January 15, 2021. It has shown a continued expansion of both funding and capture of funding across the global funding landscape in the tracker, including importantly research taking place within LMICs. The progression of funding has expanded the funding portfolio in the previously well-funded areas, in particular ‘virus natural history, transmission and diagnostics’, ‘candidate therapeutics R&D’ and ‘candidate vaccines R&D’ as well as starting to fill some of the previously less funded sub-priority areas, such as ‘supply of therapeutics’ and ethics ‘education, access and capacity building’. The latest iteration has however shown a clear plateauing of the funding response since October, with the nearly half of new funding being newly captured rather than newly funded.

We are keen for researchers, funders and policy makers to engage with these data directly for their areas of specialism and interest, through extracting the relevant data from the tracker and undertaking their own analyses to aid decision making. Given the time demands on all parties in the pandemic, we hope that the regular provision of these descriptive and thematic analyses provide broad insights to help inform the research community and improve the efficiency and effectiveness of the research response going forwards.

### Alignment of the funded research portfolio to the WHO Roadmap

Importantly, here we have aligned the funded research to the mid- long term research and innovation priorities of the WHO Roadmap, and disaggregated the data by locations and population to give a detailed picture of how the research landscape aligns to these global research priorities.

The majority of research funded aligns well to the WHO Roadmap, however, low levels of funding in the global research funding portfolio persist, specifically related to the priority areas of ‘Ethics considerations for research’ and ‘Animals and environmental’. Whilst it is not possible to tell from the funded portfolio alone, what is the appropriate level of funding for any priority area, e believe that ‘Animals and Environmental’ does represent important and real research gaps, towards which the research community should be shifting its attention. It is also important to note the intention and detail of these and all priority areas within the WHO Roadmap, where there is variability in who is best positioned to address the research sub-priorities with some clearly needing external research activity and others indicating research activity which the WHO planned to undertake directly themselves.

The lack of alignment of funded research projects to the ‘Ethics considerations for research’ priority may be one such example, as it misses the direct activity that the WHO has undertaken in to address this priority through direct research and provision of important guidance on ethical matters relating to COVID-19 ^13^ which align to the sub-priorities as well as the clear strength of ethical consideration across many research projects (which don’t have a core focus on ethics). Despite these considerations, both researchers and research funders need to pay greater attention to the prominence placed on ethical considerations for research by the WHO and ensure that further research is undertaken on those aspects outlined under the roadmap priority area explicitly for standalone ethics research.

For ‘Animals and environmental research’, again the WHO is currently undertaking direct activity in this regard (through their ongoing mission with China to identify the specific source and intermediate pathways of transmission for SARS CoV-2 into humans ^14^). However, except for a few notable projects in LMICs, the instigation of necessary broader research activity in this area, particularly looking towards gaining broader understanding of how such viruses emerge in the human population and proactive surveillance is certainly limited and needs to be expanded and longer term in nature. This research needs to be undertaken in locations where diseases are most likely to emerge, due to the nature of interactions between humans and animals ^15^, many of which are LMICs and this could therefore play an increasingly important role in the research portfolios in these locations going forward. It is also important that this research activity for this priority expands beyond the remit of the WHO and through collaborations with the UN Food and Agriculture Organisation (FAO) and the World Organisation for Animal Health (OIE) through a One Health framework ^16^.

Beyond the clear gaps at the priority level, it is inherently difficult to conclude when a particular priority area has received sufficient research funding or research projects at the grant award stage, as this is only apparent when the outcomes are achieved and it is clear that the research question has been sufficiently answered. We do however further note, that within some of the better funded WHO research priority areas, there are still certain sub-priorities which again are clear gaps. This is the case for ‘Optimal endpoints for clinical trials’ and ‘Develop core clinical outcomes to maximize usability of data across range of trials’ within the ‘Clinical characterisation and management’ priority area. This may result from the fact that these activities will be implicit but not explicit in clinical research projects; however, this in itself may indicate a clear issue where both these sub-priorities are essential for collation of results across studies and should therefore be explicit, pointing to the generally observed lack of coordination beyond a few pre-established clinical trial networks as highlighted in discussions at the recent GloPID-R Synergies Meetings ^17^. In contrast, the variability of research activity indicated within the ‘Candidate therapeutics R&D’ priority area appears to reflect the inter-dependencies of these sub-priorities rather than necessarily a gap needing immediate funding, with research into ‘Supply of therapeutics’ depending to some extent on the identification of particular safe and effective therapeutics.

For those sub-priorities where research investments have been focused there will continue to be benefits to enhanced coordination. We have already highlighted the wide range of social science research projects addressing ‘Uptake of public health measures’ and ‘Media & communication’ sub-priorities to the WHO COVID-19 Social Sciences working group. The basic virus research on ‘Diagnostic products’, ‘Virus compartments shedding and history’ and ‘Charactering immunity’ are further areas where coordination should be explored globally due to large funded research portfolios. Many of these basic virus research projects are explicitly linked to the early stages of candidate therapeutic, vaccine and diagnostic design, and as results now start to become available from late stage trials on the effectiveness of various classes of these and they are being rolled out; there is a need to refine the focus of the pipeline research more strategically to both target remaining gaps and build on emerging successes.

### Location of research

Most of the funded research projects in the tracker are located in HICs, reflecting national funding by some of the wealthiest research funders during the first phase of this pandemic, with the truly global nature of the pandemic meaning that virus was circulating in these countries to enable relevant clinical research. A large amount of research has also been funded within China, although as explained in the limitations we have not managed to incorporate this. The global distribution of funding is now shifted slightly, with recent funding calls and announcements related to LMICs and some domestic funding captured (e.g. in Brazil). There appears to be a growing recognition that context specific research is needed in LMICs ^11^, although the results presented here show only small proportional expansion of funding dedicated to context specific research priorities with an important focus on faster and easier diagnostic tools and research to understand the disease in fragile environments. There remain clear research gaps relating to health systems, optimal personal protective equipment use, health care worker support and community engagement. We published a separate detailed sub-analysis of the baseline tracker data relating to COVID 19 research in Africa, in collaboration with the African Academy of Sciences ^6^.

### Research populations

The disaggregation of research projects by populations is particularly insightful with regards to the ‘Social Sciences’ WHO Roadmap priority, but also for the ‘Clinical management’ and ‘Epidemiological studies’ priority areas. A range of vulnerable populations appear to be well represented for the social sciences including ‘minority populations’ with recent funding calls in the UK (by UKRI and National Institute for Health Research (NIHR)) having focussed on researching Black, Asian and Minority Ethnic (BAME) populations due to the emerging evidence that they are at higher risk from COVID-19 than white people. A range of health care worker populations and other frontline workers are also included in research funded which again is important due to the clear evidence on greater risks of exposure to individuals in certain occupations ^18^ in this pandemic. Children are well represented in the epidemiological studies in accordance with the prioritisation of understanding their role in transmission. The disaggregation of research by different populations can be particularly useful to policy makers and to ensuring research activity engages the necessary range of populations.

### Beyond the WHO Roadmap

Given the funded research projects within the tracker relate to disciplines beyond health (with relevance to COVID-19) it is unsurprising that several important emergent research themes identified relating to broader social sciences disciplines (policy and economy; education; logistics and food security) and also environmental research, extend beyond the priorities included in the WHO Roadmap Priorities. These all represent important areas for COVID-19 research which funders and researchers are already prioritising with research projects. The two emergent themes of mental health and digital health are however directly relevant to the health research remit and appear to have not been sufficiently covered in the WHO Roadmap document, although projects on these are being funded. The newly emerging category of research on long COVID, was not evident or anticipated as a research need at the time of development of the WHO Roadmap (although it should now inform the need for prioritisation of research including long-term follow up of cases for any newly emerging disease) ^19^. These shifting priorities emphasise the need for the WHO Roadmap to be a living roadmap.

We may also be observing the evolution of research priorities from response to recovery and expect to see further examples of this. The expansion of COVID-19 research beyond the original WHO Roadmap document illustrates the wide-reaching social, economic and cultural impacts of the pandemic. We have been collaborating with the development of the recently released UN Research Roadmap for the COVID-19 Recovery ^20^ which identifies 25 research priorities across five key areas (health systems, social protection and basic services, economic response and recovery, macroeconomic response and multilateral collaboration and social cohesion and community resilience) and future inclusion of these priorities may help categorise the many social sciences projects which fall outside the remit of the WHO roadmap.

A key strength of this tracker is its breadth and we have therefore undertaken some initial cross-cutting thematic analyses across it here to highlight additional variables that cross- cut disciplines with the inclusion of capacity strengthening, innovation, repurposed grants, modelling, cohorts, pandemic preparedness, implementation, indirect health impact, gender and capacity. The analyses on these themes will be given greater focus in ’tracker highlight’ analyses released on the tracker website ^3^.

## Conclusion

In conclusion, we have here provided a detailed review and thematic analysis across the COVID-19 funded research available and we now encourage the research community to use this and the tracker tool to support informed decision making on further research prioritisation, based on the knowledge of what research is already initiated. We encourage research funders to continue to submit their data to the tracker to ensure it can be as effective as possible.

The global research response has aligned well to the WHO Roadmap, however clear research gaps appear to remain in the portfolio relating to ‘Animals and environmental’ priority areas as well as research taking place in and relating to priorities of relevance to LMICs. Research relating to diagnostics, therapeutics and vaccines (from basic research onwards) have now all received substantial investment, across a huge number of different studies around the world. To ensure the research investments yield impact, there is now need for enhanced coordination and reprioritisation (taking stock of achievements and defining whether original priorities are still valid one year on). This is particularly important now that the initial flourish of research funding for COVID-19 appears to be plateauing.

We have also shown here the power of tracking research funding at source in real-time, which is particularly important in the fast-moving research environment created by a pandemic, but may have benefits for other global collaborative research efforts going forward. The challenge of nationally funded projects that are underpowered and therefore unable to achieve their aims, demands that researchers and funders be much more strategic going forwards to efficiently and effectively advance knowledge within epidemics and pandemics. Tools such as this tracker can facilitate global collaboration and solidarity to maximise the efficiency and impact of research funding.

### Limitations of findings and challenges

To the best of our knowledge we have compiled the most comprehensive database of funded COVID-19 research. We are however very mindful of its inherent limitations and the difficulties in gaining a fully comprehensive picture in what is a truly global research effort to a global pandemic. One main limitation is the absence of commercial research data making inferences on gaps in the vaccine and therapeutics portfolios difficult (this is lacking due to associated intellectual property restrictions). This tracker however has rich data on the early stage development research for those same priorities which is valuable for public funder coordination efforts and enables thematic analyses across disciplines. Another limitation is the fact that few funders to date have shared data on repurposed grants or grants for institutional funding which may have been used for COVID-19 related research.

We are also aware of several funders across wider geographies and disciplines, from whom we have not yet been able to incorporate data. We call here for further research funders (especially within LMICs) to submit their data to make this tracker and associated analyses more accurate to improve the ongoing coordination and help focus limited resources.

The alignment of research in this tracker to the priorities outlined in the WHO Roadmap also has its challenges, given the Roadmap was produced at speed by drawing together findings from different working groups operating in different ways. The resulting priorities are unsurprisingly imbalanced with some covering much broader research areas than others and with not all sub-priorities intended to be addressed by newly funded research. We have tried to account for this in the discussion of the results here. Another limitation of these priorities and indeed any priorities in a pandemic is their limited temporal nature. The WHO Roadmap priorities that we have mapped here, although named mid- to long-term priorities, were identified by world experts in February 2020, at a time when the majority of cases of COVID-19 were still in China and a pandemic had not yet been declared. We will therefore be updating our mapping as soon as any new version of the WHO Roadmap is released as research priorities have evolved with increasing knowledge.

### Sustainability and future work

This living mapping review will be updated on a quarterly basis for the duration of the COVID CIRCLE initiative. Future planned work includes incorporation of any new priorities or sub-priorities from any revision of the WHO Roadmap. Given the tracker contains a broad range of research relating to COVID-19 (beyond health research) and the evolution towards longer term thinking around research priorities, we are also discussing incorporating coding to the UN COVID Recovery Research Roadmap ^20^ and are in discussions with the team who have developed this.

## Data availability

### Underlying data

The continuingly updated data related to this study are openly available in the ‘COVID-19 Research Project Tracker by UKCDR & GloPID-R’ at https://www.ukcdr.org.uk/funding-landscape/covid-19-research-project-tracker/.

Harvard Dataverse: Replication Data for ‘A living mapping review for COVID-19 funded research projects: six-month update’ https://doi.org/10.7910/DVN/WG7YNH ^12^

This project contains the following underlying data:

- Replication data for ‘A living mapping review for COVID-19 funded research projects: six- month update’ (2021-03-15)

### Extended data

Figshare: Extended data for ‘A living mapping review for COVID-19 funded research projects: six-month update. https://doi.org/10.6084/m9.figshare.14216291.v1 ^7^

This project contains the following extended data:

- Ext data 1 COVID19 Research Project Tracker_Template_ Norton *et al.* August 2020.xlsx (Template spreadsheet)
- Ext data 2 grant sources Norton *et al.* Dec 2020.docx (43.01 kB)
- Ext data 3 WHO priorities Norton *et al.* August 2020.docx (WHO COVID-19 research priorities)
- Ext data 4 African and LMIC research priorities Norton *et al.* August 2020.docx (List of African and LMIC research priorities)
- 20201118 - Figure 1 (PRISMA Flow).docx
- 202001104 Figures (2, 4, 5, 10, 12) and table 3.docx
- 20201104 - Figures (3, 6 & 11).pptx
- Figures 7, 8 and 9.docx

### Reporting guidelines

Figshare: PRISMA checklist for ‘A living mapping review for COVID-19 funded research projects: six-month update. https://doi.org/10.6084/m9.figshare.14216291.v1 ^7^

Data are available under the terms of the Creative Commons Zero "No rights reserved" data waiver (CC0 1.0 Public domain dedication).
