# Supplementary material for: A living mapping review for COVID-19 funded research projects: final (27 month) update
Source: Wellcome Open Res. 2023 Jul 26;5:209. Originally published 2020 Sep 8. [Version 10] doi: 10.12688/wellcomeopenres.16259.10 (PMC7579366; doi:10.12688/wellcomeopenres.16259.10)
Supplement: Peer review history

# Peer review: Reviewer response for version 10

**DOI:** 10.21956/wellcomeopenres.21597.r63901

**Published:** 2023-08-03

**Authors:** David Vaughn

**Affiliations:** 1 Bill and Melinda Gates Foundation, Seattle, WA, USA

No further comments.

Are the rationale for, and objectives of, the Systematic Review clearly stated?

Yes

Is the statistical analysis and its interpretation appropriate?

Not applicable

Are sufficient details of the methods and analysis provided to allow replication by others?

Yes

Are the conclusions drawn adequately supported by the results presented in the review?

Yes

Reviewer Expertise:

NA

I confirm that I have read this submission and believe that I have an appropriate level of expertise to confirm that it is of an acceptable scientific standard.

# Peer review: Reviewer response for version 8

**DOI:** 10.21956/wellcomeopenres.20529.r53344

**Published:** 2022-11-23

**Authors:** David Vaughn

**Affiliations:** 1 Bill and Melinda Gates Foundation, Seattle, WA, USA

Approve version 8.

Are the rationale for, and objectives of, the Systematic Review clearly stated?

Yes

Is the statistical analysis and its interpretation appropriate?

Not applicable

Are sufficient details of the methods and analysis provided to allow replication by others?

Yes

Are the conclusions drawn adequately supported by the results presented in the review?

Yes

Reviewer Expertise:

NA

I confirm that I have read this submission and believe that I have an appropriate level of expertise to confirm that it is of an acceptable scientific standard.

# Peer review: Reviewer response for version 6

**DOI:** 10.21956/wellcomeopenres.19909.r51745

**Published:** 2022-07-29

**Authors:** David Vaughn

**Affiliations:** 1 Bill and Melinda Gates Foundation, Seattle, WA, USA

No additional comments.

Are the rationale for, and objectives of, the Systematic Review clearly stated?

Yes

Is the statistical analysis and its interpretation appropriate?

Not applicable

Are sufficient details of the methods and analysis provided to allow replication by others?

Yes

Are the conclusions drawn adequately supported by the results presented in the review?

Yes

Reviewer Expertise:

NA

I confirm that I have read this submission and believe that I have an appropriate level of expertise to confirm that it is of an acceptable scientific standard.

# Peer review: Reviewer response for version 5

**DOI:** 10.21956/wellcomeopenres.19586.r49399

**Published:** 2022-03-22

**Authors:** David Vaughn

**Affiliations:** 1 Bill and Melinda Gates Foundation, Seattle, WA, USA

No new comments.

Are the rationale for, and objectives of, the Systematic Review clearly stated?

Yes

Is the statistical analysis and its interpretation appropriate?

Not applicable

Are sufficient details of the methods and analysis provided to allow replication by others?

Yes

Are the conclusions drawn adequately supported by the results presented in the review?

Yes

Reviewer Expertise:

Vaccine development.

I confirm that I have read this submission and believe that I have an appropriate level of expertise to confirm that it is of an acceptable scientific standard.

# Peer review: Reviewer response for version 4

**DOI:** 10.21956/wellcomeopenres.18739.r44742

**Published:** 2021-07-01

**Authors:** David Vaughn

**Affiliations:** 1 Bill and Melinda Gates Foundation, Seattle, WA, USA

No further comments.

Are the rationale for, and objectives of, the Systematic Review clearly stated?

Yes

Is the statistical analysis and its interpretation appropriate?

Not applicable

Are sufficient details of the methods and analysis provided to allow replication by others?

Yes

Are the conclusions drawn adequately supported by the results presented in the review?

Yes

Reviewer Expertise:

Vaccine development.

I confirm that I have read this submission and believe that I have an appropriate level of expertise to confirm that it is of an acceptable scientific standard.

# Peer review: Reviewer response for version 3

**DOI:** 10.21956/wellcomeopenres.18438.r43370

**Published:** 2021-04-01

**Authors:** David Vaughn

**Affiliations:** 1 Bill and Melinda Gates Foundation, Seattle, WA, USA

No further comments.

Are the rationale for, and objectives of, the Systematic Review clearly stated?

Yes

Is the statistical analysis and its interpretation appropriate?

Not applicable

Are sufficient details of the methods and analysis provided to allow replication by others?

Yes

Are the conclusions drawn adequately supported by the results presented in the review?

Yes

Reviewer Expertise:

Vaccine development.

I confirm that I have read this submission and believe that I have an appropriate level of expertise to confirm that it is of an acceptable scientific standard.

# Peer review: Reviewer response for version 2

**DOI:** 10.21956/wellcomeopenres.18093.r41885

**Published:** 2020-12-21

**Authors:** Peter Smith

**Affiliations:** 1 MRC Tropical Epidemiology Group, London School of Hygiene and Tropical Medicine, London, UK

Are the rationale for, and objectives of, the Systematic Review clearly stated?

Yes

Is the statistical analysis and its interpretation appropriate?

Not applicable

Are sufficient details of the methods and analysis provided to allow replication by others?

Yes

Are the conclusions drawn adequately supported by the results presented in the review?

Yes

Reviewer Expertise:

NA

I confirm that I have read this submission and believe that I have an appropriate level of expertise to confirm that it is of an acceptable scientific standard.

# Peer review: Reviewer response for version 1

**DOI:** 10.21956/wellcomeopenres.17862.r40581

**Published:** 2020-10-21

**Authors:** Peter Smith

**Affiliations:** 1 MRC Tropical Epidemiology Group, London School of Hygiene and Tropical Medicine, London, UK

This assembly of the database on which this “systematic review” is based clearly represents a major effort. The database is likely to be most useful to funders, who will wish to avoid supporting research that is unnecessarily duplicative and it will also be of value to researchers seeking to identify others who are working in a similar area or to identify research areas that seem to have been relatively neglected. The authors have gathered data on research supported by 25 funders, mostly based in high income countries. How complete this is, even for these funders is difficult to judge, but based on the projects supported by each funder, there seems to be a strong bias towards UK funders. It is surprising, for example, to see the US NIH having supported less than 25% of the number supported by UKRI. A notable deficiency in the database, acknowledged by the authors, is the lack of data from China, where there has been substantial research on SARS-CoV-2, and LMICs in general (e.g. Brazil, India). However, it is understandable that there are challenges in accessing comprehensive information from these sources. Another challenge is that most, but not all, of the information presented relates to the number of projects supported rather than the magnitude of the support. The data base is also missing information from private companies, including pharma companies, where much research on vaccines, therapeutics and diagnostics is likely to be supported. It is also unclear whether the databases of registered clinical trials have been trawled to identify ongoing research. For these reasons, I am not convinced that it is appropriate to label the paper as a systematic review as it seems to be a review only of what has been accumulated in the database to date.

 I recognize that the database is early in its life, but as an ongoing check on completeness it may be useful, in the future, to link published papers on SARS-CoV-2 to the database to see what is not being picked up in the database, acknowledging that it is not infrequently difficult to link a particular paper to a particular research grant.

 In the review, projects in the database are linked to WHO Blueprint list of research priorities defined in February 2020. This helps identify areas where there are possible funding gaps. However, there are some limitation in using the data base for this purpose. For example, the authors note that there are clear gaps in support for 'optimal endpoints for clinical trials and core clinical outcomes’ and on ‘ethical considerations for research’. Both of these areas have had considerable attention, the former in the context of treatment and vaccine trials (the authors acknowledge the problems in identifying research that may be embedded in other research undertakings) and much of the ethical work that has been done has not required specific funding.

 Despite some of the deficiencies identified above, the generation of the database has been a valuable undertaking, and as more funders come on board it will become even more useful. Similarly, the ongoing systematic review will give a useful summary of what is in the database and will help identifying trends in funding and areas where support has been lacking.

Are the rationale for, and objectives of, the Systematic Review clearly stated?

Yes

Is the statistical analysis and its interpretation appropriate?

Not applicable

Are sufficient details of the methods and analysis provided to allow replication by others?

Yes

Are the conclusions drawn adequately supported by the results presented in the review?

Yes

Reviewer Expertise:

NA

I confirm that I have read this submission and believe that I have an appropriate level of expertise to confirm that it is of an acceptable scientific standard.

# Response

**Published:** 2020-11-30

**Authors:** Alice Norton

**Affiliations:** University of Oxford, UK

Thank you very much for your helpful comments on our Baseline paper, we are pleased that you believe that this has been a valuable undertaking. We have now updated the review with the latest data in the tracker up to the 15 ^th^ October and have also taken that opportunity to address some of your comments within the new version of the paper. This continues (in our view) to be the most comprehensive data set on research funded on COVID-19 with now over five thousand projects from 72 funders (all categorised to the WHO R&D Blueprint). We have now provided further details relating to magnitude of funding commitment in addition to numbers of projects in the figures relating to funders and agree that this helps with interpretation. We have noted and agree with the limitations that you highlight regarding the challenges in accessing the full breadth of global research data in this field and we have already clearly acknowledged these limitations. We also agree that the ‘systematic’ terminology may not therefore be fully appropriate for this endeavour and have now instead re-categorised this as a living mapping review. We also agree with your points on the particular sub-priorities in the roadmap which may have received attention outside of newly funded or even repurposed grants, and we have further clarified our discussion of these. We are continuing to expand the global representation of funders in the database and are exploring connections with both clinical trial registrations and outputs (although neither of these are straightforward). We hope that you find this latest version of the paper of renewed interest and relevance.

# Peer review: Reviewer response for version 1

**DOI:** 10.21956/wellcomeopenres.17862.r40348

**Published:** 2020-09-21

**Authors:** David Vaughn

**Affiliations:** 1 Bill and Melinda Gates Foundation, Seattle, WA, USA

This is a well-written report by the UKCDR and GloPID-R on their living systematic review (Project Tracker) of COVID-19 R&D including 1858 projects supported by 25 funders across 102 countries as of 15 July 2020 aligning said R&D efforts with the WHO global research roadmap for COVID-19 R&D published in March 2020. The tracker also identifies research objectives not included in the WHO roadmap (seven new areas) that may influence planning for future pandemics (including future WHO roadmaps). A spin-off of the effort (COVID CIRCLE) focuses on R&D in resource-limited settings.

 The Tracker allows the global health community to assess the COVID-19 research portfolio to identify gaps (Animal and environmental research? Clinical trial endpoints and outcomes?) and to reduce duplication (more than 300 vaccines being advanced?); reduction of excessive duplication is important given limited global R&D resources.

 The authors are transparent concerning the limitations of the effort. The data is skewed to GloPID-R members. It is heavily UKRI biased. The US NIH, in particular, seems under-represented. Re-purposed funded research may be missed. There is limited data from a number of governments (e.g., China as noted by authors) and private sources (e.g., companies, private foundations such as the BMGF, and ethics think tanks). This publication may encourage more LMICs to participate in the accounting.

 Is it possible to comment on measured or perceived impacts? Who uses? Is there documentation of times accessed? Is it timely information if the figures are updated only every three months? The group should conduct a survey at the end of the calendar year to see if funders changed direction based upon this effort. Has there been a reduction of duplication? Or, will this be a retrospective of what happened with little real-time impact on what was happening? Shifting of funding to LMICs is one positive example of impact. Again, we will need a later assessment to build upon this baseline review focusing on impact on the pandemic response. Has the scope been too broad?

Are the rationale for, and objectives of, the Systematic Review clearly stated?

Yes

Is the statistical analysis and its interpretation appropriate?

Not applicable

Are sufficient details of the methods and analysis provided to allow replication by others?

Yes

Are the conclusions drawn adequately supported by the results presented in the review?

Yes

Reviewer Expertise:

Vaccine development.

I confirm that I have read this submission and believe that I have an appropriate level of expertise to confirm that it is of an acceptable scientific standard.

# Response

**Published:** 2020-11-30

**Authors:** Alice Norton

**Affiliations:** University of Oxford, UK

Thank you very much for your very helpful comments on our baseline paper. We are delighted to now provide a three-month update paper in which the number of projects and funders has greatly expanded over the interval (to over 5 thousand projects from 72 funders). We aim to continue to achieve further geographical cover through ongoing efforts to raise the profile of the tracker and source data from further funders. Thank you for your suggestion regarding inclusion of data on use of these data and perceived impact, we have now included reference to this in this three-month update. We will also be undertaking a retrospective consultation on the influence of this work as part of the learning element of COVID CIRCLE in early 2021. We hope that you find this latest version of the paper of renewed interest and relevance and we would also like to highlight the more frequent updates and highlights available on the funding tracker website.
