# Supplementary material for: A living mapping review for COVID-19 funded research projects: one year update
Source: Wellcome Open Res. 2022 Mar 22;5:209. Originally published 2020 Sep 8. [Version 5] doi: 10.12688/wellcomeopenres.16259.5 (PMC7579366; doi:10.12688/wellcomeopenres.16259.5)
Supplement: Peer review history

# Response

**Published:** 2020-11-30

**Authors:** Alice Norton

**Affiliations:** UK Collaborative on Development Research, UK

Thank you very much for your helpful comments on our Baseline paper, we are pleased that you believe that this has been a valuable undertaking. We have now updated the review with the latest data in the tracker up to the 15 ^th^ October and have also taken that opportunity to address some of your comments within the new version of the paper. This continues (in our view) to be the most comprehensive data set on research funded on COVID-19 with now over five thousand projects from 72 funders (all categorised to the WHO R&D Blueprint). We have now provided further details relating to magnitude of funding commitment in addition to numbers of projects in the figures relating to funders and agree that this helps with interpretation. We have noted and agree with the limitations that you highlight regarding the challenges in accessing the full breadth of global research data in this field and we have already clearly acknowledged these limitations. We also agree that the ‘systematic’ terminology may not therefore be fully appropriate for this endeavour and have now instead re-categorised this as a living mapping review. We also agree with your points on the particular sub-priorities in the roadmap which may have received attention outside of newly funded or even repurposed grants, and we have further clarified our discussion of these. We are continuing to expand the global representation of funders in the database and are exploring connections with both clinical trial registrations and outputs (although neither of these are straightforward). We hope that you find this latest version of the paper of renewed interest and relevance.

# Response

**Published:** 2020-11-30

**Authors:** Alice Norton

**Affiliations:** UK Collaborative on Development Research, UK

Thank you very much for your very helpful comments on our baseline paper. We are delighted to now provide a three-month update paper in which the number of projects and funders has greatly expanded over the interval (to over 5 thousand projects from 72 funders). We aim to continue to achieve further geographical cover through ongoing efforts to raise the profile of the tracker and source data from further funders. Thank you for your suggestion regarding inclusion of data on use of these data and perceived impact, we have now included reference to this in this three-month update. We will also be undertaking a retrospective consultation on the influence of this work as part of the learning element of COVID CIRCLE in early 2021. We hope that you find this latest version of the paper of renewed interest and relevance and we would also like to highlight the more frequent updates and highlights available on the funding tracker website.
